# Enhancing thymic function improves T-cell reconstitution and immune responses in aged mice

**DOI:** 10.1371/journal.pbio.3003283

**Published:** 2025-07-28

**Authors:** Abigail Morales-Sánchez, Marieke Lavaert, Melanie S. Vacchio, Gustavo Ulises Martinez-Ruiz, Daniel Egbase, Yongge Zhao, Ross Lake, Masaki Ishikawa, Fatima Zohra Braikia, Dragana Jankovic, Ranjan Sen, Rémy Bosselut, Avinash Bhandoola, Jennifer E. Cowan

**Affiliations:** 1 National Cancer Institute, National Institutes of Health, Bethesda, Maryland, United States of America; 2 Children’s Hospital of Mexico Federico Gomez, Mexico City, Mexico; 3 Research Division, Faculty of Medicine, National Autonomous University of Mexico, Mexico City, Mexico; 4 Division of Immunity and Infection, University College London, London, United Kingdom; 5 National Institute of Aging, National Institutes of Health, Bethesda, Maryland, United States of America; 6 National Institute of Allergy and Infectious Diseases, National Institutes of Health, Bethesda, Maryland, United States of America; Children's Hospital of Philadelphia and The University of Pennsylvania School of Medicine, UNITED STATES OF AMERICA

## Abstract

Age-related thymic involution leads to diminished output of naïve T-cells. While this process is suggested to increase the risk of disease severity in the elderly following infection, direct evidence is lacking. We developed two mouse models that allow us to experimentally prevent or reverse thymic involution. Constitutive Myc expression in thymic epithelial cells (TEC) of middle-aged mice enhanced thymic function, and increased numbers of peripheral naïve CD4 and CD8 T-cells. Inducible Myc expression reversed age-related thymic involution and partially recovered peripheral naïve T-cell numbers. Importantly, improving thymic function in these settings preserved T-cell-dependent antibody responses and significantly reduced T-cell-associated mortality after infection with *Toxoplasma gondii*. Improved thymic function also rebalanced age-associated alterations in the Treg pool, and mitigated loss of the transcriptional Th1 signature in aged conventional T-cells. Our findings support the value of TEC-focused thymic regeneration strategies for enhancement of T-cell-mediated immunity in the elderly.

## Abstract 2

Age-related thymic decline impairs T-cell immunity and increases infection risk in the elderly. This study shows that enhancing thymic function in aged mice restores naïve T cells and improves immune responses, thus supporting thymic regeneration in aging.

## Introduction

Age-related thymus involution is an evolutionarily conserved process characterized by a gradual reduction in size and function [1]. Thymic involution initiates during early life and continues throughout adulthood [2–5]. It leads to a diminished population of naïve T-cells in peripheral tissues and contraction of the T-cell receptor (TCR) repertoire [6–8]. While a decrease in early thymic progenitors accompanies aging in mice [9], thymus involution is likely to be primarily triggered by dysfunction of thymic epithelial cells (TEC) [6,9]. These TEC are specialized cells that guide the development of functionally competent and self-tolerant T-cells [10]. Cortical (c)TEC are essential for selecting thymocytes expressing functional TCRs. Medullary (m)TEC play a key role in central tolerance by the elimination of self-reactive T-cells and the differentiation of nonconventional T-cells, such as regulatory T-cells (Tregs) [11]. Changes in TEC composition and numbers during aging alter their function [6,12,13]. These include age-related changes to mTEC and their capacity to present self-antigens and mediate central tolerance [6,14]. Thus, changes in the TEC compartment during thymic involution are well described. Moreover, there is a strong correlation between loss of thymic function and progressive immune decline across the life course. However, although thymus involution has been suggested as a mechanism that underpins decreased immune responsiveness and increased susceptibility to infections in the elderly [15], direct evidence to demonstrate this is lacking.

Because of the significant immunological consequences of TEC disruptions, considerable attention has been directed toward discovering approaches to mitigate such disruptions. Pharmacological interventions such as administration of interleukin-7 (IL-7), keratinocyte growth factor (KGF), and degarelix (for sex steroid ablation), each enhanced T-cell lymphopoiesis [16–19]. In addition, genetic manipulation aimed at preventing the age-associated downregulation of Forkhead Box N1 (*Foxn1*) and fibroblast growth factor (*FgF*)*21* protected against age-related thymic atrophy [20,21]. Inducible expression of *FoxN1* conditionally in TEC partially reconstituted the size of the aged thymus and populations of splenic naïve T-cells [22]. However, whether enhancing thymic function mitigates immune deficiencies observed in the elderly remains uncertain. KGF administration to 15-month-old mice restored thymic cellularity and augmented peripheral T-cell numbers and T-cell-dependent antibody production; however, protection against infection was not examined [23]. The administration of KGF or sex steroid ablation in aged (18–22 months) mice did not increase the number of naïve T-cells and recent thymic emigrants in secondary lymphoid organs and did not protect against infection with West Nile virus [19]. Thus, further investigations are warranted to elucidate how enhanced thymopoiesis may benefit the peripheral T-cell compartment and improve the immune function in the elderly.

We previously demonstrated that Myc regulates a transcriptional program in fetal TEC that controls their rapid expansion in numbers during development. Sustained, enforced expression of Myc in TEC maintains this rapid expansion, increasing total thymic size in adulthood [24]. In the present study, we investigated the impact of thymus involution on T-cell-mediated immunity. We generated constitutive (MycTg) and inducible (iMycTg) transgenic mouse models. Prolonged Myc expression in TEC of middle-aged MycTg mice caused thymic hyperplasia, whereas inducible expression of Myc in aged iMycTg mice reversed age-related thymic involution. Furthermore, thymic growth using either continuous or inducible expression of Myc in TEC improved the T-cell-associated mortality of older mice after infection with *Toxoplasma gondii*. Thymic hyperplasia prevented the decline in naïve CD4 and CD8 T-cells in the periphery observed with aging, and enhanced B-cell-mediated responses. Reversing involution partially recovered naïve T-cells in the periphery. Alterations to peripheral Treg cells were also detectable in both mouse models, along with expanded TCR repertoire diversity and transcriptional alterations to T-cells following challenge. Thus, TEC mediated prevention or reversal of thymic involution enhances T-cell mediated immunity during aging.

## Results

### Prevention of thymus involution overcomes the T-cell-associated mortality of older mice infected with *T. gondii*

**Fig 1:**
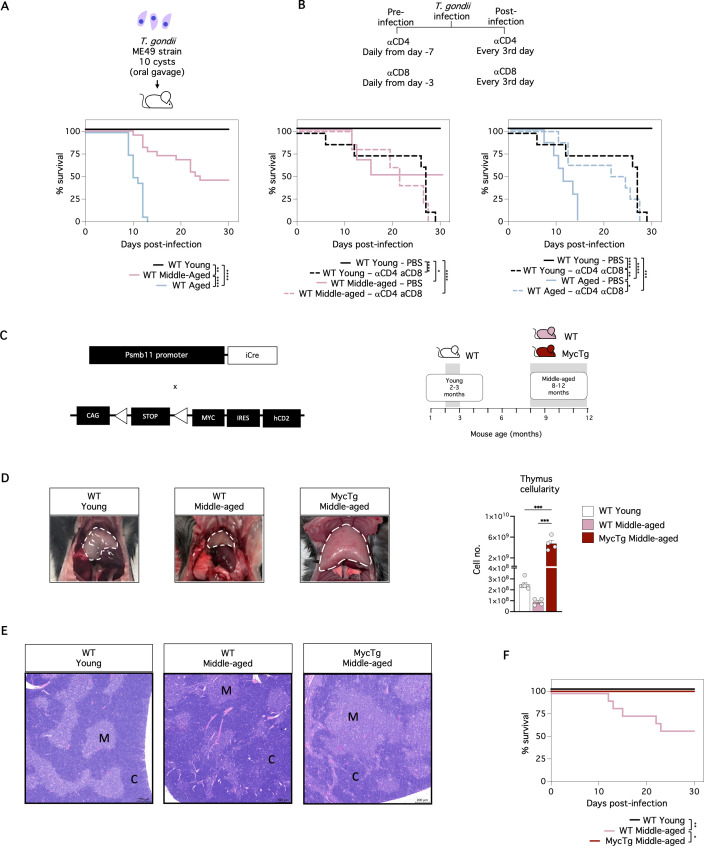
Enhanced thymic function overcomes T-cell-mediated mortality in middle-aged individuals infected with *T. gondii.* Mice were infected with 10 cysts of *T. gondii* (strain ME49) and survival was followed up for 30 days. (**A)** Kaplan–Meier plots showing the survival of WT young (2–3 months of age) vs. WT middle-aged (8–12 months of age) vs. WT aged mice (15-18 months of age) (*n* = 11–24 mice per group from three independent experiments). (**B)** The schematic on the top shows the αCD4 and αCD8 administration protocol. Kaplan–Meier plots showing the survival of WT young vs. WT middle-aged mice (left) (*n* = 5-8 mice per group from two independent experiments) and WT young vs. WT aged mice (right) (*n* = 7–8 mice per group from two independent experiments), receiving depleting antibodies (dotted lines) or PBS (solid lines). (**C)** A simplified schematic of the genetic mouse model and the experimental groups. β5t-Cre mice [30] were crossed to Myc^stopFL^ mice [31] to generate the constitutive Myc transgenic model (MycTg, left). A timeline showing the age intervals of mice selected for experimental groups (right). (**D)** Representative pictures (left) and total thymus cell counts (right) of WT young, WT middle-aged, and MycTg middle-aged mice. (**E)** H&E staining of thymus sections from 9-week-old WT young (left), 6-month-old WT (middle), and 6-month-old MycTg (right) mice. (C: cortex, M: medulla). Scale bar = 200 µm. **(F)** Kaplan–Meier plots showing the survival of WT young vs. WT middle-aged vs. MycTg middle-aged mice (*n* = 6–12 mice per group from two independent experiments). *p < 0.05, **p < 0.01, ***p < 0.001, and ****p < 0.0001. The data underlying this figure can be found in S7 File.

Previous studies have reported that certain strains of *T. gondii* can be lethal to aged mice, while causing mild effects in young mice [25,26]. We infected wild type (WT) young (2–3 months of age), WT middle-aged (8–12 months of age), and WT aged (15–18 months of age) mice with 10 cysts of *T. gondii.* We followed up on their survival 30 days after infection. All young mice survived, whereas approximately half of middle-aged mice died, and all aged mice succumbed within the initial 2 weeks (Fig 1A). Since T-cells play a crucial role in host defense against *T. gondii* [27,28] and age-related thymic involution is suggested to compromise T-cell immunity [15], we hypothesized that the higher mortality of older mice could potentially be linked to changes in T-cells with aging.

To investigate the contribution of T-cells to the mortality of older mice, we depleted T-cells by administering anti(α)-CD4 and α-CD8 treatments [27] to young WT and middle-aged WT mice and aged WT mice before and after infection (Fig 1B). All mice treated with αCD4 and αCD8 antibodies succumbed within 30 days after parasite inoculation (Fig 1B). However, mice treated with αCD4 and αCD8 antibodies exhibited similar kinetics in mortality regardless of their age, with most deaths occurring after day 20 post-infection. Interestingly, the antibody treatment significantly delayed mortality in WT aged mice compared to WT aged mice treated with PBS (Fig 1B, right). Thus, T-cell depletion eliminated the age-dependent rapid mortality following *T. gondii* infection. These findings indicate that T-cells play a pivotal role in age-associated mortality during *T. gondii* infection.

We aimed to assess the contribution of thymus involution to the age-associated mortality of old mice infected with *T. gondii.* We previously demonstrated that sustained, enforced expression of Myc from the lox-Stop-lox-controlled Rosa26:Myc locus in TEC using a Foxn1Cre increased total thymic size in adulthood [24]. In the present study, we generated a transgenic mouse model (MycTg) in which enforced Myc expression was driven by β5t-Cre, specifically in TEC. β5t is a TEC proteasome subunit encoded by the *Psmb11* gene [29] that in the thymus is specifically expressed by TEC progenitors that give rise to cTEC and the majority of mTEC [30]. Myc^stopFL^ mice were previously generated by targeting the *ROSA26* locus with the human *Myc* cDNA, which is preceded by a *loxP* flanked STOP cassette, and controlled by the CAG promoter [31]. The Myc transgene includes a signaling-deficient truncated version of human (h)CD2 as a reporter (Fig 1C, left and Materials and methods), allowing for the identification of cells expressing the transgene. The frequency of hCD2 expression ranged consistently between 90% and 100% across all TEC subsets (S1A Fig), indicating effective and widespread activation of the Myc transgene within the total TEC population.

We examined MycTg middle-aged, WT middle-aged, and WT young mice (Fig 1C, right). MycTg mice showed a dramatic increase in thymus cellularity (Fig 1D). Although enforced Myc expression enhanced cell numbers, we did not see extracapsular diffusion or capsule effraction (Fig 1D). In addition, histological analyses revealed no obvious morphological alterations to the architecture of the MycTg middle-aged thymi compared to the WT middle-aged control (Fig 1E), consistent with our previous data in young adult mice [24]. Our results indicate that constitutive, enforced Myc expression in TEC prevented the reduction of thymus size associated with thymic involution and instead caused thymic hyperplasia.

We investigated the impact of enhanced thymic function on the survival of middle-aged mice to *T. gondii* infection. Because MycTg mice do not survive past 9–12 months of age, likely due to excessive thymic size causing cardio-respiratory failure [32,33], we were only able to use middle-aged MycTg mice for these studies. Remarkably, preventing thymus involution rescued middle-aged mice from mortality, with a 100% survival rate until the end of the experiment (Fig 1F). Taken together, our results reveal the influence of age on *T. gondii* infection outcomes and establish the pivotal role of T-cells in age-related mortality.

### Myc-mediated TEC expansion prevents age-associated alterations to peripheral T-cells

**Fig 2:**
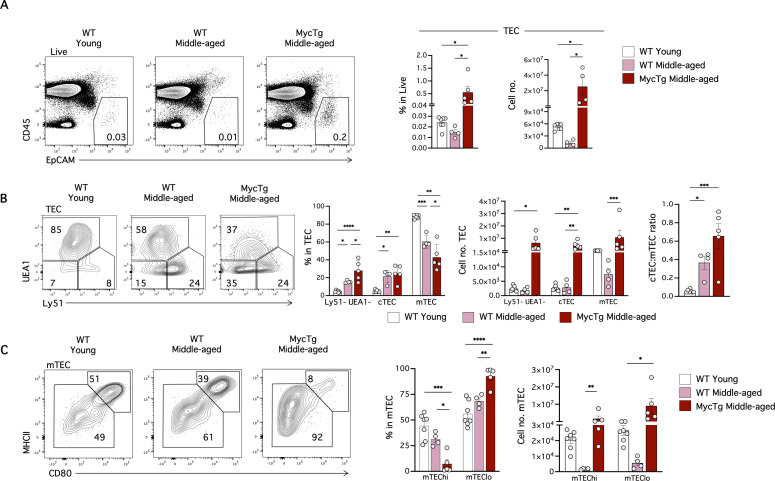
Enforced expression of Myc in TEC. **(A)** Representative FACS plots showing total thymic epithelial cells (TEC, CD45^−^ EpCAM^+^). Bar graphs displaying TEC percentages and absolute cell numbers in WT young (2–3 months of age), WT middle-aged (8–12 months of age), and MycTg middle-aged (8–12 months of age) mice. **(B)** Representative FACS plots showing Ly51 and UEA1 staining on TEC. Bar graphs indicate percentages and absolute cell numbers of the Ly51^−^ UEA1^−^ TEC, Ly51^+^ UEA1^−^ (cTEC) and Ly51^−^ UEA1^+^ (mTEC) subsets within the total TEC, and the cTEC:mTEC ratio. **(C)** Representative FACS plots showing MHCII and CD80 expression within mTEC. Bar graphs indicate percentages and absolute cell numbers of MHCII^hi^ CD80^hi^ (mTEC^hi^), MHCII^lo^ CD80^lo^ (mTEC^lo^) subsets within mTEC. *p < 0.05, **p < 0.01, ***p < 0.001, and ****p < 0.0001. The data underlying this figure can be found in S7 File.

We next examined the impact of enforced Myc expression on TEC and peripheral T-cell populations in middle-aged mice. MycTg mice showed a significantly elevated frequency and absolute numbers of TEC (CD45^−^ EpCAM^+^) (Fig 2A). There were no significant alterations in the percentage of cTEC (Ly51^+^ UEA1^−^), but there was an increased percentage of the Ly51^−^ UEA1^−^ TEC subset and a reduction in the percentage of mTEC (Ly51^−^ UEA1^+^) between WT middle-aged and MycTg middle-aged mice (Fig 2B). Moreover, the ratio of cTEC:mTEC was altered in the middle-aged groups compared to the WT young (Fig 2B). Finally, we observed an alteration in the mTEC^hi^ (MHC-II^hi^ CD80^hi^) to mTEC^lo^ (MHC-II^lo^ CD80^lo^) ratio in MycTg middle-aged mice (Fig 2C).

**Fig 3:**
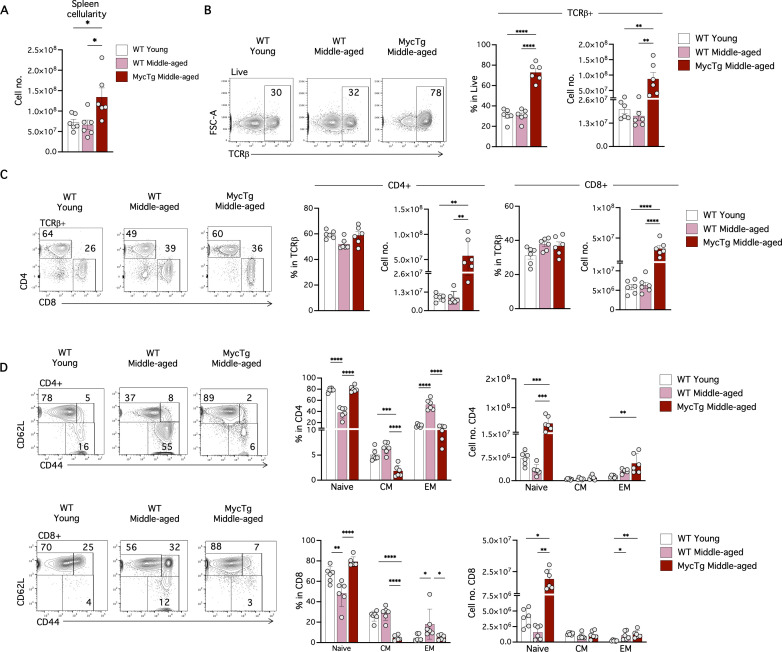
Enforced Myc expression in TEC prevents the age-associated decline in peripheral naïve CD4 and CD8 T-cells. **(A)** Total spleen cellularity of WT young (2–3 months of age), WT middle-aged (8–12 months of age), and MycTg middle-aged (8–12 months of age) mice. **(B)** Representative FACS plots of TCRβ expression on total splenic cells. Bar plots showing the percentages and absolute numbers of TCRβ^+^ cells. **(C)** Representative FACS plots and bar plots of the CD4 and CD8 T-cells (pre-gated in TCRβ^+^ cells) frequencies and absolute numbers. **(D)** CD62L and CD44 expression on TCRβ^+^ CD4 (top) and TCRβ^+^ CD8 (bottom) T-cells. Bar plots depicting percentages and quantitation of CD62L^+^ CD44^−^ (naive), CD62L^+^ CD44^+^ central memory (CM), and CD62L^−^ CD44^+^ effector memory (EM) in WT young, WT middle-aged, and MycTg middle-aged mice. *p < 0.05, **p < 0.01, ***p < 0.001, and ****p < 0.0001. The data underlying this figure can be found in S7 File.

Thymic involution results in reduced production of T-cells [34] and a diminished pool of naïve T-cells in the periphery [19,22]. We therefore investigated peripheral T-cells of MycTg mice, examining two distinct secondary lymphoid organs—the spleen and inguinal lymph nodes (iLN). MycTg middle-aged mice exhibited a significant increase in spleen and iLN cellularity with higher numbers of total T-cells (TCRβ^+^), CD4, CD8, naïve CD4 and naïve CD8 T-cell subsets (Figs 3A–3D and S2A–S2D). Interestingly, MycTg mice maintained frequencies of naïve CD8 and CD4 T-cells akin to those observed in WT young mice (Figs 3D and S2D). Frequencies of central memory (CM) and effector memory (EM) subsets of CD4 and CD8 T-cells were reduced as a consequence of thymic hyperplasia. However, because of the overall increase in T-cell numbers in MycTg mice, the absolute numbers of CM and EM CD4 and CD8 T-cells were unchanged in the spleen compared to WT middle-aged mice (Fig 3D) and even increased in the iLN (S2D Fig).

**Fig 4:**
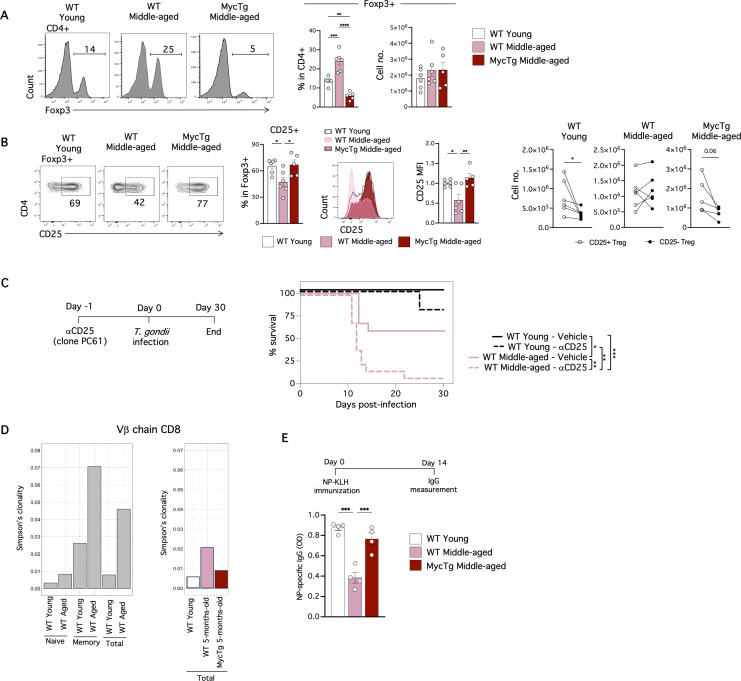
Myc-mediated TEC expansion prevents age-associated alterations to peripheral T-cells, including Treg accumulation, reduced TCR repertoire diversity, and T-cell-mediated antibody responses. **(A)** Representative Foxp3 staining in TCRβ^+^CD4 T-cells in the spleens of WT young (2–3 months of age), WT middle-aged (8–12 months of age), and MycTg middle-aged (8–12 months of age) mice. **(B)** Representative FACS plots of the CD25 expression within Foxp3^+^ Treg. Bar plots showing the CD25^+^ Treg frequency. FACS histograms and bar plots showing the median fluorescence intensity (MFI) of CD25 pre-gated in Foxp3^+^ Treg. Dot plots showing the absolute number of CD25^+^ vs. CD25^−^ Treg per mouse in WT middle-aged and MycTg middle-aged relative to WT young mice. **(C)** The schematic of the αCD25 administration protocol (left). Kaplan–Meier plot showing the survival curve of WT young vs. WT middle-aged mice that received αCD25 depleting antibodies or vehicle (right) (*n* = 9–13 mice from two independent experiments). **(D)** Simpson’s clonality index of CD8 T-cells isolated from the spleens of WT young and WT aged mice (left) or WT young, WT middle-aged and MycTg middle-aged (right). **(E)** NP-specific IgG quantification in serum at day 14 after immunization of the indicated mouse groups. *p < 0.05, **p < 0.01, ***p < 0.001, and ****p < 0.0001. The data underlying this figure can be found in S7 File.

An increased percentage of Foxp3^+^ Treg cells is observed during aging [35,36]. Interestingly, MycTg middle-aged mice did not present with an increased percentage of Foxp3^+^ Treg (Figs 4A and S2E). The absolute number of this population remained unchanged in the spleen (Fig 4A) but was significantly augmented in the iLN of middle-aged MycTg mice (S2E Fig). A decrease of the CD25^hi^ Treg cells was observed in WT middle-aged mice but not in MycTg middle-aged mice (Fig 4B).

Treg cells play a pivotal role in balancing protective and pathological responses during *T. gondii* infection [37,38]. To investigate whether the decrease in the CD25^hi^ Treg population in middle-aged mice (Fig 4B) was associated with mortality due to *T. gondii* infection, we investigated the effects of reducing the CD25^hi^ Treg cells [39] further in middle-aged mice during *T. gondii* challenge. Young and middle-aged mice were treated with α-CD25-depleting antibodies one day before *T. gondii* infection. On day 9 post-infection both α-CD25-treated groups had reduced frequencies and numbers of total Treg cells and CD25^hi^ Treg (S3A Fig). The mortality rate of WT middle-aged mice significantly increased upon CD25^hi^ Treg depletion, with the survival of only one mouse, compared to the survival of the majority of young α-CD25 treated mice (Fig 4C). These data demonstrate an age-dependent need for CD25^hi^ Treg to control this parasitic infection and suggest the gradual decline of CD25^hi^ Treg cells with age contributes to the mortality of older mice infected with *T. gondii*.

As mice age, a reduction in TCR diversity is evident in peripheral T-cells [40]. We investigated whether enhanced immune function could prevent or reverse the decline in the TCR repertoire. We performed β-chain sequencing exclusively on CD8 T-cells, to remove the effects of Treg cell contamination in the CD4 total T-cell population. We compared the Simpson’s clonality index (in which higher values indicate reduced clonal diversity) of WT CD8 T-cells isolated from the spleens of young and aged mice and observed a reduced clonal diversity in aged mice (Fig 4D, left). To explore if this expansion was driven by the overrepresentation of CD44^hi^ antigen-experienced T-cells in the aged sample, we subdivided T-cells based on CD44 expression. The greatest expansion in the Simpson’s clonality between young and aged samples was observed in the CD44^hi^ memory subset. However, a modest increase was also observed in the CD44^lo^ naïve aged T-cell fraction (Fig 4D, left). Thus, the overall expansion in clonality and the restriction in diversity of the TCR repertoire with age are driven by the expanded CD44^+^ memory population, yet naïve CD8 T-cells isolated from aged individuals may also have restricted TCR diversity. To examine if enhancing thymic function counteracted the increase in TCR clonality observed in total T-cells from older mice, we performed the same β-chain sequencing on CD8 T-cells from 5-month-old MycTg mice. The results demonstrated a preserved TCR diversity of CD8 T-cells comparable to a WT young control (Fig 4D, right), thus confirming a contribution of thymic involution in the increased clonality observed with aging.

Finally, we assessed if TEC expansion-mediated prevention of thymus involution altered the functional capacity for T-cells to mediate antibody responses. Previous studies have shown that B-cells from aged mice do not display intrinsic defects in responding to immunization [41,42]. Instead, the age of CD4 T-cell donors influences germinal center formation and antibody production in response to nitrophenyl acetyl conjugated to keyhole limpet hemocyanin (NP-KLH) in reconstituted SCID mice [43]. To assess if humoral responses are enhanced if thymic involution is prevented, we immunized WT young, WT middle-aged, and MycTg middle-aged mice with T-cell dependent NP-KLH antigen. NP-specific IgG was quantified at day 14 after immunization. Interestingly, MycTg mice immunized with the T-dependent NP-KLH antigen did not have the decline in antibody production seen in WT middle-aged mice (Fig 4E). This demonstrates that enhanced thymic function in middle age can enhance humoral protection, producing comparable levels of T-cell-dependent antibody responses to WT young mice following immunization. Furthermore, this suggests that the thymic involution process may contribute to the diminished T-cell-dependent antibody production observed in aged mice.

### Reversion of thymus involution delays the mortality of aged mice infected with *T. gondii*

**Fig 5:**
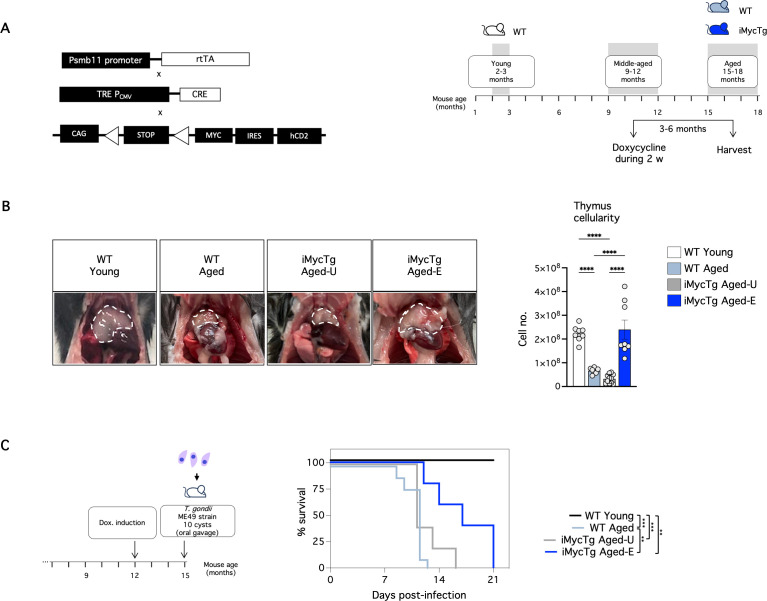
Thymus regeneration delays the mortality of aged mice infected with *T. gondii.* **(A)** A simplified schematic illustrating the experimental model and the experimental groups used. β5t-rtTA × tetO-Cre mice [44] were crossed to Myc^stopFL^ [31] to generate iMycTg mice (left). Mice at 9–12 months of age were given doxycycline in drinking water for 2 weeks and used in experiments 3–6 months later (averages and ranges of the elapsed time post-doxycycline are given below for each experiment). WT aged (15–18 months of age) mice were given doxycycline as iMycTg mice. WT mice at 2–3 months of age were also included as young controls. **(B)** Representative pictures (left) and total thymus cellularity (right) of the WT young, WT aged, iMycTg-U (unenlarged), and iMycTg-E (enlarged) mice. Mice were analyzed on average 4.3 months after induction (range: 3.5–5.1 months). **(C)** Schematic of doxycycline induction and *T. gondii* infection (left). Kaplan–Meier plot showing the survival of WT young vs. WT aged vs. iMycTg aged-U vs. iMycTg aged-E mice (right) after infection. The experiment was concluded on day 21 post-infection as iMycTg-E aged mice were moribund. Mice were analyzed on average 3.1 months after induction (range: 3–3.6 months) (*n* = 5–10 mice per group). *p < 0.05, **p < 0.01, ***p < 0.001, and ****p < 0.0001. The data underlying this figure can be found in S7 File.

We established a transgenic model, Myc-hCD2stopFL × β5t-rtTA × tetO-Cre [44], allowing inducible, enforced expression of Myc conditionally in TEC (Fig 5A, left). Transgenic mice aged 9–12 months received doxycycline for two weeks and were harvested 3–6 months later (see figure legends for the specific time point per experiment). Doxycycline-treated littermates (WT aged) and WT young mice served as controls (Fig 5A, right). The iMycTg aged mice exhibited thymus regrowth, with cell numbers akin to young WT mice (Fig 5B), indicating a successful reversal of the age-related thymic involution. We defined thymus regrowth as thymic cellularity greater than 100 × 10^6^ cells (1.5 times the average size of the WT aged thymus). Intriguingly, the thymus regenerated in only a subset (6 out of 17 tested mice, or 35%) of iMycTg mice (iMycTg aged-enlarged (E) hereafter) and remained unenlarged (iMycTg aged-U hereafter) and with similar cellularity as the WT aged controls in the remainder (Fig 5B). The reason for this variability in thymus regeneration across iMycTg mice remains elusive, as no associations with mouse sex or time post-doxycycline administration were identified. Even more puzzling is the finding that the hCD2 reporter is comparably expressed by TEC isolated by the iMycTg aged-E and iMycTg aged-U mice (S4A Fig). This suggests that inconsistent regrowth is not due to a technical issue with the model. Instead, Myc is induced efficiently within approximately 60% of TEC but, for unknown reasons, the thymus does not rebound in some mice (S4A Fig).

*T. gondii* infection induced thymic atrophy in young and middle-aged mice, as evidenced by a severe reduction in thymic cellularity at days 3 and 9 post-infection (S4B Fig), consistent with previous findings [45]. As previously mentioned, only a subset of induced iMycTg mice showed thymus regrowth (Fig 5B). We screened aged iMycTg mice using thymic ultrasound before infection, revealing a significant correlation between thymic volume or area and thymus cellularity. Thymic ultrasound guided the selection of mice predicted to have an enlarged thymus (volume > 10 mm^3^ corresponding to >100 × 10^6^ cells) (Figs 4D and S4C). Subsequently, iMycTg aged mice predicted to have an enlarged thymus or an unenlarged thymus, along with young and aged controls were infected. Interestingly, while iMycTg aged-E mice still experienced mortality, this was delayed compared to iMycTg aged-U mice and age-matched controls (Fig 5C). Particularly striking was the survival of all iMycTg aged-E mice during the acute phase of the infection, during which both WT aged mice and iMycTg aged-U mice succumbed. This outcome suggests that the dysregulation of T-cell responses, which drives rapid mortality in old mice around 9–14 days post-infection did not occur in the iMycTg aged-E mice. Our transgenic model, thus, demonstrates the potential of inducible Myc expression in TEC to reverse age-related thymic involution and improve the survival of infected aged mice.

### Inducible expression of Myc in TEC promotes recovery of some age-associated alterations to peripheral T-cells

**Fig 6:**
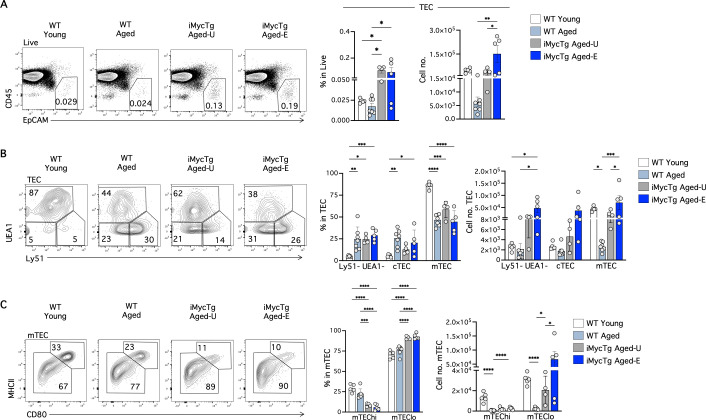
Inducible enforced expression of Myc in TEC. **(A)** Representative FACS plots showing total thymic epithelial cells (TEC, CD45^−^ EpCAM^+^) in live cells. Bar graphs indicate TEC percentages and absolute cell numbers in WT young (2–3 months of age), WT aged (15–18 months of age), iMycTg aged-U (unenlarged, 15–18 months of age), and iMycTg aged-E (enlarged, 15–18 months of age) mice. **(B)** Representative FACS plots showing Ly51 and UEA1 detection pre-gated in TEC. Bar graphs indicate the absolute cell numbers and percentages of Ly51^−^ UEA1^−^, Ly51^+^ UEA1^−^ (cTEC), and Ly51^−^ UEA1^+^ (mTEC) subsets within total TEC. **(C)** Representative FACS plots showing MHCII and CD80 expression within mTEC. Bar graphs indicate percentages and absolute cell numbers of MHCII^hi^ CD80^hi^ (mTEC^hi^) and MHCII^lo^ CD80^lo^ (mTEC^lo^) subsets within mTEC. Mice were analyzed on average 3.9 months after induction (range: 3.5–4.8 months). *p < 0.05, **p < 0.01, ***p < 0.001, and ****p < 0.0001. The data underlying this figure can be found in S7 File.

We then analyzed the effects of the inducible Myc transgene on TEC populations and peripheral T-cells, that resulted in an improved survival of the mice infected with *T. gondii*. TEC frequency and number significantly increased in iMycTg aged-E mice compared to WT aged controls (Fig 6A). There were no significant alterations in the percentage of cTEC, mTEC or Ly51^−^ UEA1^−^ TEC (relative to total TEC) subsets between WT aged and iMycTg aged-E mice (Fig 6B). However, further analysis revealed that the mTEC^hi^ population displayed a significantly lower percentage in iMycTg aged-E mice than young and aged controls (Fig 6C), mimicking the TEC changes observed in middle-aged MycTg mice (Fig 2C). Interestingly, both the constitutive MycTg middle-aged (Fig 2B) and the inducible iMycTg aged-E models (Fig 6B) displayed characteristics of aging TEC, with expansion of the Ly51^−^ UEA1^−^ EpCAM^+^ population [46]. Overall, there were no differences in the percentage of total TEC or any TEC subset between iMygTg-U and iMycTg-E. However, the cellularity of TEC, mTEC, and mTEC^lo^ was higher in iMycTg-E compared to iMygTg-U mice (Fig 6A–6C).

**Fig 7:**
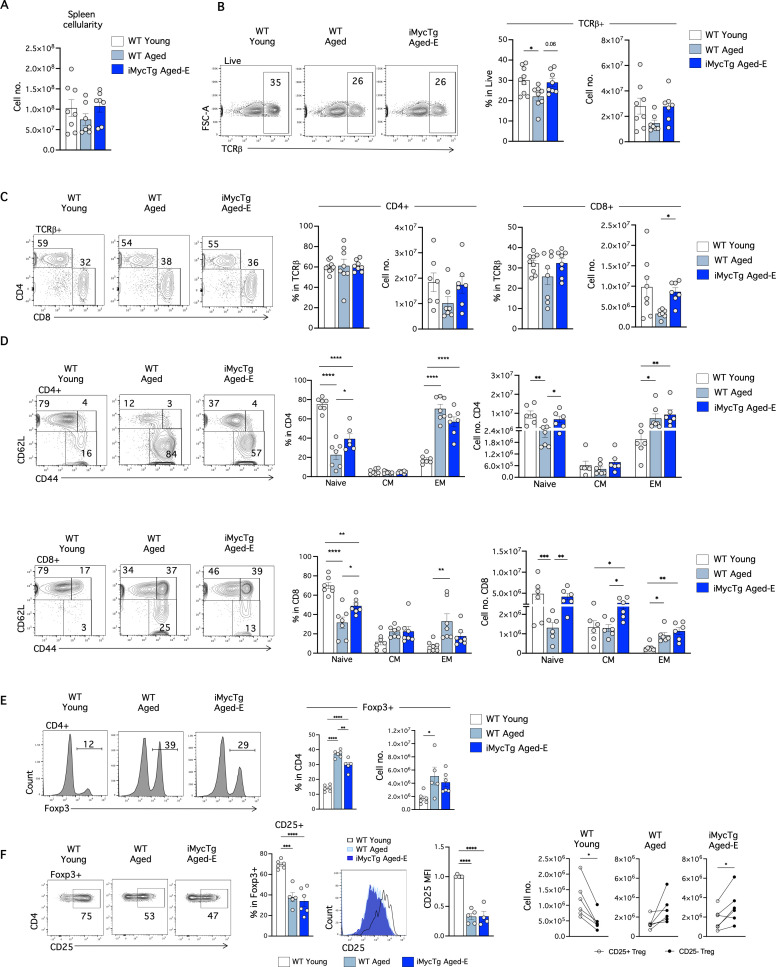
Thymus regeneration promotes naïve T-cell recovery in iMycTg aging-E mice and a partial decrease in Treg frequencies. **(A)** Total spleen cellularity of WT young (2–3 months of age), WT aged (15–18 months of age), and iMycTg aged-E (enlarged) (15–18 months of age) mice. **(B)** Representative FACS plots of the expression of TCRβ in the total splenic cells. Bar plots showing the percentages and absolute numbers of TCRβ^+^ cells. **(C)** Representative FACS plots and bar plots of the CD4 and CD8 T-cell (pre-gated in TCRβ^+^) frequencies and absolute numbers in the indicated mouse groups. **(D)** CD62L and CD44 expression on TCRβ^+^ CD4 (top) and TCRβ^+^ CD8 (bottom) T-cells. Bar plots depicting percentages and quantitation of CD62L^+^ CD44^−^ (naive), CD62L^+^ CD44^+^ central memory (CM), and CD62L^−^ CD44^+^ effector memory (EM) T-cells in the indicated mouse groups. **(E)** Representative Foxp3 staining on CD4 splenic T-cells. Bar plots show the percentage and quantification of Foxp3^+^ Treg cells. **(F)** FACS plots and bar plots of the CD25^+^ cells frequency within Foxp3^+^ Treg in WT young, WT aged and iMycTg aged-E. Histograms and bar plots in the middle show the median fluorescence intensity (MFI) of CD25 pre-gated in FoxP3^+^ Treg, relative to WT young mice. Dot plots on the right show the absolute number of CD25^+^ and CD25^−^ Treg cells per mouse in the indicated mouse groups. Mice were analyzed on average 4.5 months after induction (range: 3.4–6.0 months). *p < 0.05, **p < 0.01, ***p < 0.001, and ****p < 0.0001. The data underlying this figure can be found in S7 File.

We investigated the impact of thymus regeneration on peripheral T-cells of iMycTg aged-E mice, focusing only on the subset of mice that showed regrowth. Spleen cellularity showed no significant differences between young and aged mice (Fig 7A). Notably, thymus rejuvenation allowed recovery in spleen of the percentage of T-cells (TCRβ^+^) that had significantly declined with age (Fig 7B). Similarly, the decline in numbers of TCRβ^+^ CD4 and TCRβ^+^ CD8 T-cells in the spleen of aged mice was partially reversed in iMycTg aged-E mice (Fig 7C). Remarkably, iMycTg aged-E mice exhibited a significantly higher percentage and number of naïve CD4 and CD8 T-cells than WT aged mice, while the EM subset remained comparable between the two groups (Fig 7D). Thus, thymus regeneration in iMycTg aged-E mice resulted in a larger pool of naïve T-cells. Consistent with past work [19] and distinct from our findings in the spleen, we saw minimal alterations in the iLN due to thymus regrowth (S5A–S5E Fig).

We next determined the effect of thymic regrowth on Foxp3^+^ Treg with aging in the spleen. The Foxp3^+^ Treg population was 2.5-fold higher in WT aged mice than in WT young controls. Interestingly, iMycTg aged-E mice showed a significant reduction in the percentage of Treg compared to WT aged mice (Fig 7E). However, the absolute number of Treg cells was increased in both aged groups (Fig 7E). Thymic regeneration did not restore the frequencies of CD25^hi^ Treg in iMycTg aged-E mice (Fig 7F). Although the Foxp3^+^ Treg to Foxp3^−^ T-conventional ratio within the CD4 T-cells was gradually augmented during aging and diminished during thymic rejuvenation (Fig 7E), the numbers of Treg and their age-associated phenotypical characteristics were maintained relatively constant (Fig 7F).

**Fig 8:**
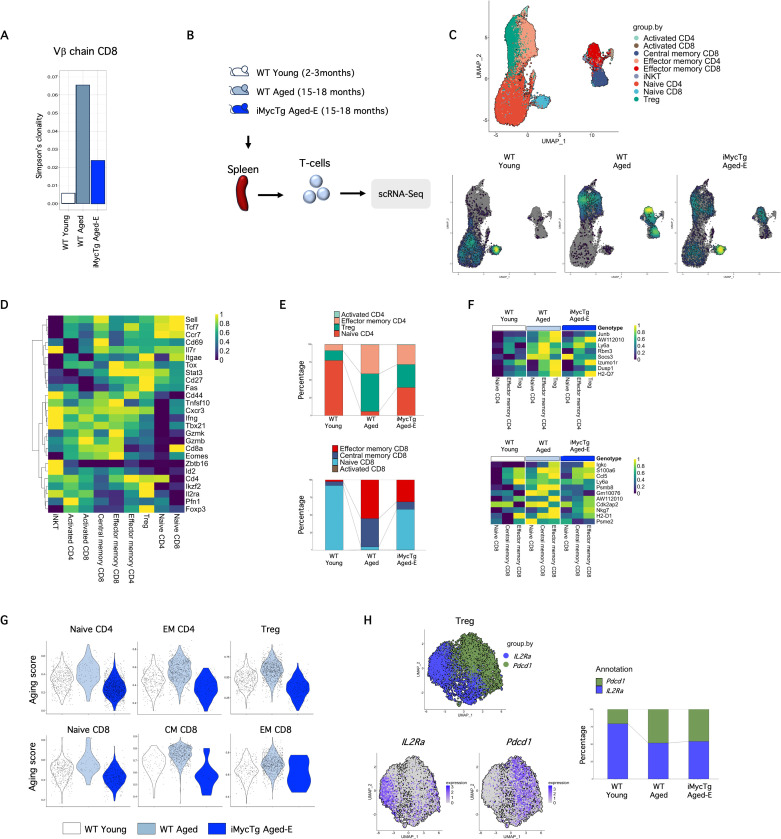
Thymus regeneration increases TCR repertoire diversity and partially restores transcriptional signatures of T-cells during aging. **(A)** Simpson’s clonality index of CD8 T-cells isolated from the spleens of WT young (2–3 months of age), WT aged (15–18 months of age), and iMycTg aged-E (enlarged) (15–18 months of age) mice. Mice were analyzed 5.9 months after induction. **(B)** Overview illustrating single-cell (sc)-RNA Seq experiment on T-cells. Data from this study were integrated with two other publicly available datasets [47,48] to ensure robustness, but only cells from this study are displayed in the figure. **(C)** Annotated UMAP of T-cells isolated from WT young, WT aged, and iMycTg aged-E mice at steady state. Annotated populations are shown at the top, and cell densities per experimental group are shown at the bottom. **(D)** Heatmap of the annotated populations. **(E)** Bar plots showing the abundance of CD4 and CD8 T-cells subsets identified by scRNA-Seq. **(F)** Heatmaps on genes upregulated in aged CD4 (top) and CD8 (bottom) T-cells used to make an aging score (see Materials and methods) shown in **G**. **(G)** Violin plots showing aging scores for naïve, effector memory (EM) and regulatory T-cells (Treg) CD4 T-cells (top), and for naïve, CM and EM CD8 T-cells (bottom). **(H)** UMAPs (left) and bar plot (right) showing Treg (Foxp3^+^) cells grouped by their expression of *Il2ra* (encoding CD25) and *Pdcd1* (encoding PD1). Mice were analyzed 5.9 months after induction. The data underlying this figure can be found in S7 File.

Finally, to examine if thymic regrowth in aged mice was sufficient to restore TCR repertoire diversity, we performed β-chain sequencing on CD8 T-cells isolated from iMycTg aged-E mice. The Simpson clonality index revealed that restoring thymic function in the aged counteracted the reduced diversity observed in the aged-matched control (Fig 8A). Thus, confirming a contribution of thymic involution to the reduced TCR diversity observed with aging, which can be restored with TEC-mediated regenerative strategies.

### Transcriptome analysis at steady state reveals T-cell dynamics and transcriptional signatures during aging and thymus regeneration

To further investigate T-cell dynamics during aging and restored thymus function, we performed single-cell RNA sequencing (scRNA-Seq) on WT young, WT aged, and iMycTg aged-E mice, where each sample represents one biological replicate per experimental condition (Fig 8B). We obtained data from splenic CD4 T-cells and CD8 T-cells (sorted as live, CD19^−^ TCRβ^+^ CD4^+^ or CD8^+^). To increase the statistical power and robustness of our analyses, we integrated our data with two previously published scRNA-Seq datasets of aged CD4 and CD8 T-cells [47,48] (see Materials and methods). Integrated data was used to perform clustering analyses but only our own data sets are displayed in the figures, unless otherwise specified. Clustering revealed three major CD4 T-cell subpopulations designated as naïve, EM, and Treg, and three major CD8 T-cell subpopulations designated as naïve, CM, and EM (Fig 8C), based on their gene expression profiles (Fig 8D). Small clusters of activated CD4 T-cells, activated CD8 T-cells, and iNKT cells were also identified (Fig 8C). In our datasets, we observed a single γδ T-cell, which we excluded from further analyses. Naïve CD4 and CD8 T-cells exhibited high expression levels of *Sell* (encoding CD62L), *Tcf7*, and *Ccr7*. EM CD4 cells expressed high levels of *CD44* and *Cxcr3,* while Tregs were identified by the expression of *Foxp3* and the highest level of *Il2ra* (encoding CD25). CM CD8 T-cells expressed *Sell* along with activation markers like *CD44* and *CD69*. Finally, EM CD8 T-cells showed high expression of *Eomes*, *Tox*, and *Gzmk* (Fig 8D).

As expected, the WT aged sample showed a reduced proportion of naïve T-cells and an increased abundance of the EM and CM compartments compared to WT young (Fig 8C, bottom and 8E). Consistent with flow data, naïve CD4 and CD8 T-cell abundance was partially recovered, and Treg expansion was counteracted in the iMycTg aged-E sample (Fig 8C, bottom and 8E). Thus, iMycTg aged-E T-cells reflected a nuanced cellular landscape, showing enrichment of memory cells alongside a substantial presence of naïve cells (Fig 8C, bottom and 8E). By comparing gene expression profiles between young and aged T-cells using our data set, Elyahu and colleagues [47] and Mogilenko and colleagues [48], we identified a set of 8 genes upregulated in total CD4 and a set of 11 genes upregulated in total CD8 T-cells in aged mice (Fig 8F). From aged CD4 T-cells, we identified *AW112010* and Izumo 1 receptor (*Izumo1r),* both of which have been previously associated with T-cell aging [47], as well as suppressor of cytokine signaling 3 (*SOCS3*), known to be upregulated in aging processes of the muscle and nervous system [49–51]. Additionally, other identified markers in aged CD4 T-cells were *Junb*, *Ly6a*, *Rbm3*, *Dusp1*, and *H2-Q7* (Fig 8F). In aged CD8 T-cells, we identified *S100a6* encoding a calcium-binding protein. *S100a6* has previously been shown to be upregulated in in vitro stimulated T-cells from old mice compared to young mice [52]. *Ccl5* (encoding CCL5/RANTES) was shown to be upregulated in CD8 T-cells from 20-month-old compared with 3-month-old mice [52]. Additionally, its plasma levels have been shown to increase with aging in humans [53]. *Nkg7* has been identified as crucial for CD8 T-cell functions, including cytotoxicity and antitumor T-cell immunity [54–56]; however, it has not been linked to T-cell aging. Similarly, *Igkc*, *Psmb8*, *Psme2*, *Gm10076*, *Cdk2ap2* and *H2-D1* represent new age markers in CD8 T-cells. *Ly6a* and *Aw112010* were found to be upregulated in both aged CD4 and CD8 T-cells (Fig 8F).

We subsequently employed the sets of genes upregulated in aged CD4 and CD8 T-cells to generate aging scores for CD4 and CD8 T-cells (see Materials and methods), enabling the assessment of aging-related gene expression patterns within individual T-cells from in-house generated and publicly available data [47,48]. Notably, iMycTg aged-E T-cells showed a significantly reduced aging score *versus* WT aged cells for all CD4 and CD8 T-cell clusters (Fig 8G). Moreover, using only our data set, we generated aging scores from differentially expressed genes (DEG) upregulated (S6A Fig) or downregulated (S6B Fig) between WT young and WT aged samples per individual T-cell cluster and applied the scores to the iMycTg aged-E equivalent clusters. All iMycTg aged-E T-cell subsets had lower scores for genes upregulated with age, and higher scores for genes downregulated with age compared to the WT aged subsets. This trend was particularly clear in the naïve CD4 and naïve CD8 subsets (S6A and S6B Fig). Genes down-regulated with age included Cluster of Differentiation 28 (*Cd28*), CD3 epsilon subunit of the T-cell receptor complex (*Cd3e)*, Tyrosin-protein kinase (*Lck*), protein tyrosine phosphatase receptor type C (*Ptprc*), and thymocyte selection associate (*Themis*) (S1 File) and are involved in T-cell receptor signaling (S2 File). A full list of DEG per cluster is listed in S1 File, and all biological processes associated with them are listed in S2 and S3 Files. Collectively, both approaches generating aging scores suggest that T-cells isolated from iMycTg aged-E mice are more ‘transcriptionally youthful’ than their aged-matched counterparts. These transcriptional differences need further exploration to identify possible functional consequences of this restored transcriptional youth.

Although flow cytometric analysis confirmed iMycTg aged-E mice do not present with diminished numbers of Treg or higher frequencies of CD25^hi^ Treg subsets compared to WT aged mice (Fig 7E and 7F), the aging score of the Treg cluster for iMycTg aged-E samples was reduced compared to WT aged (Fig 8G). We, therefore, delved deeper into possible transcriptional differences within the Treg subsets. We identified two transcriptionally distinct clusters defined by the expression of *Il2ra* (encoding CD25) or *Pdcd1* (encoding PD1) (Fig 8H, left). We observed a decrease in the frequency of *the Il2ra* expressing Tregs and an increase in the frequency of the *Pdcd1* expressing population with aging and an equal contribution of iMycTg aged-E and WT aged Treg frequencies in the aged samples (Fig 8H, right). Taken together, these results strengthen the finding that thymic regeneration does not diminish the accumulated CD25^lo^ Treg population observed in aging.

### Transcriptome analysis reveals an altered Th1 signature in aged mice infected with *T. gondii*

Given the T-cell-specific role of age-associated mortality to *T. gondii* infection, we evaluated if a proportion of *T. gondii*-specific CD4 T-cells was altered in the spleen of MycTg middle-aged and iMycTg aged-E mice compared to age-matched controls. I-A(b) *T. gondii* MHC-II tetrameter (TGME49 Class II I-Ab tetramer loaded with the AS-15 peptide)-binding cells were barely detected day 7 after infection at any age (S7A Fig). By day 9, WT young and middle-aged mice both had increased frequency and number of MHC-II Tet^+^ cells compared to day 7. Furthermore, at day 9 the MycTg middle-aged mice had equal frequencies and numbers to WT young and WT middle-aged mice (S7B Fig, top), whereas WT aged mice and iMycTg aged-E mice displayed decreased frequency and number of tetramer binding cells (S7B Fig, bottom). This suggests reversion of thymic involution did not rescue the age-associated decline in parasite-specific T-cell frequencies and numbers for this specific *T. gondii* epitope. Nonetheless, the use of only one *T. gondii* epitope cannot dismiss the possibility that other parasite-specific T-cells may have altered numbers and contribute to the improved survival of transgenic mice.

We next quantified the levels of a set of cytokines in the serum of iMycTg aged-E and control mice on day 9 after *T. gondii* inoculation (see Materials and methods). Some cytokines, such as IFN-γ, TNF-α, CXCL9, CXCL10, CXCL2, and IL-6 were highly upregulated in the serum of infected mice. However, iMycTg aged-E mice did not differ from WT aged mice in serum levels for measured cytokines (S8A Fig), thus providing no evidence of possible dysregulated cytokine production that may mediate accelerated death following *T. gondii* infection in WT aged mice compared to iMycTg aged-E mice. Nevertheless, our studies measured serum levels of these molecules and did not address whether alterations of cytokines were evident in tissues.

**Fig 9:**
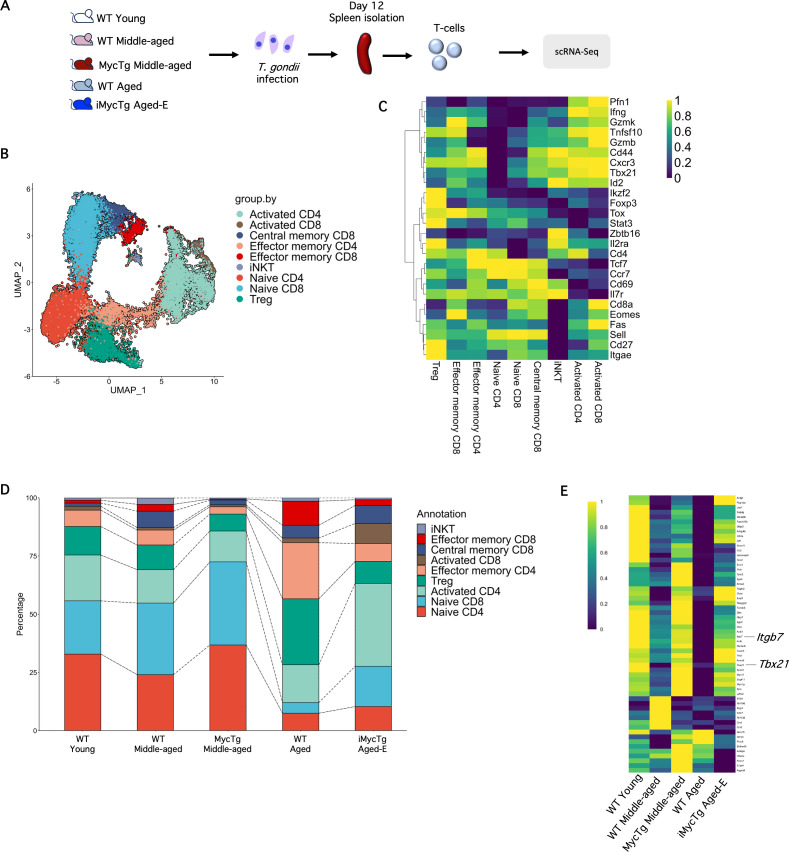
Transcriptome analysis reveals an altered Th1 signature in aged mice infected with *T. gondii.* **(A)** Overview of the scRNA-Seq experiment; WT young (2–3 months of age), WT middle-aged (8–12 months of age), MycTg middle-aged, WT aged (15–18 months of age), and iMycTg aged-E (enlarged) (15–18 months of age) mice were infected with 10 cysts of *T. gondii*. T-cells were isolated from the spleen on day 12 after infection. A UMAP (**B**) and a heatmap (**C**) of the annotated CD4 and CD8 T-cell populations. **(D)** Bar plot showing the abundance of the annotated CD4 and CD8 T-cell populations. **(E)** Heatmap showing the expression of 42 of the total Th1-associated genes [63] within the activated CD4 T-cell cluster. Mice were analyzed 5.9 months after induction. The data underlying this figure can be found in S7 File.

To gain more insight into the mechanism behind the observed enhanced survival in aged mice with restored thymic function, we conducted scRNA-Seq analysis on total T-cells (sorted as live, CD19^−^ TCRβ^+^ CD4^+^, or CD8^+^) collected on day 12 after *T. gondii* infection. We included samples from WT young, WT middle-aged, MycTg middle-aged, WT aged, and iMycTg aged-E mice, and each sample represents one biological replicate per experimental condition (Fig 9A). CD4 T-cells were categorized into distinct subsets, including naïve, EM, and Tregs, while CD8 T-cells were classified as naïve, CM, and EM. Additionally, we identified a significant cluster of activated CD4 T-cells and a smaller cluster of activated CD8 T-cells (Fig 9B). Activated CD8 T-cells exhibited higher expression levels of Granzyme B (*Gzmb*) and Fas Cell Death Receptor (*Fas*) (Fig 9C). Activated CD4 T-cells upregulated *IFNg* and *Cxcr3*, genes associated with a Th1 response [57–59]. Activated CD4 also showed elevated expression of *Gzmb*, a marker of cytotoxic CD4 T-cells [60] and *Id2*, associated with the CD4 T-cell immune response during autoimmune encephalomyelitis (Fig 9C) [61].

An enrichment of naïve CD4 and CD8 T-cells was still evident in the transgenic mice, despite the expected differentiation of T-cells from a naïve to a memory phenotype during infection (Fig 9D). This result was confirmed by flow cytometry (Figs S9A and S9B). Interestingly, while the frequencies of activated CD4 and CD8 T-cells were slightly reduced in both groups of middle-aged samples compared to young, the iMycTg aged-E sample showed an important raise in activated CD4 and CD8 T-cells compared to the age-matched control sample (Fig 9D).

Toxoplasmosis triggers a robust Th1 response [62]. We used a previously published Th1 gene signature [63] to analyze Th1-associated gene expression changes over aging and improved thymic function. A heatmap displaying the expression level of all the genes included in the analyses is shown in S10A Fig. Interestingly, more than 40 Th1-associated genes were downregulated with aging in the activated CD4 T-cell cluster but were restored in the MycTg middle-aged and iMycTg aged-E mice (Fig 9E). For instance, expression of *Tbx21* (encoding T-bet), a master regulator of Th1 fate [64–66] and *Itgb7*, an extracellular protein involved in the trafficking of Tregs into the gut [67,68], was diminished in WT middle-aged and WT aged mice and recovered in MycTg middle-aged and iMycTg aged-E mice (Fig 9E). These findings underscore significant alterations in the transcriptional profile of T-cells over aging and emphasize the critical role of thymic function in supplying T-cells capable of mounting a robust Th1 response for host defense against *T. gondii*.

Finally, to more broadly explore transcriptional changes occurring within each T-cell subset during a *T. gondii* infection and to examine if they are altered with age or thymic function, we generated infection scores. We generated a list of all DEG that were upregulated and downregulated during infection in young mice for each individual T-cell cluster (S10B and S10C Fig). We did not see clear patterns of infection scores that were altered by age or genotype. However, we did see partial restoration of the “infection score—genes up with infection” in the naïve CD4 and Treg clusters of the MycTg and iMycTg-E mice. A full list of DEG per cluster is listed in S4 File and all biological processes associated with them are listed in S5 and S6 Files.

## Discussion

The contribution of thymic involution to the age-associated changes to T-cells and dysregulated immune responses in the elderly remains unclear. This hinders the development of potential thymus targeting strategies to improve age-related immune decline, and is at least in part due to the limited availability of models to temporally restore thymic function in old age and assess its consequences [19–21,23]. Here, we developed two mouse models to experimentally prevent or reverse the process of involution by forcibly expressing Myc exclusively in TEC in either a constitutive or inducible manner. Sustained Myc expression in TEC enhanced thymic size and function and consequently resulted in high numbers of naïve CD4 and CD8 T-cells in the spleen and iLN of aging mice, with levels above those of WT young controls. Furthermore, this thymic hyperplasia enhanced humoral protection in middle-aged mice, producing T-cell-dependent antibody responses at comparable rates to WT young mice following immunization. The inducible expression of Myc in TEC of aged mice restored thymic size comparably to WT young mice, but interestingly, not in all mice. When regrowth did occur (iMycTg aged-E mice), the numbers of naïve CD4 and CD8 T-cells in the spleen partially recovered. Moreover, when challenged with the parasitic infection *T. gondii*, both the MycTg middle-aged and iMycTg aged-E mice models displayed improved disease outcomes compared to their age-matched controls. Thus, TEC-mediated enhancement of thymic function in aging can offer immunological protection to the host.

The age-associated mortality of *T. gondii* infection in mice was reported over 40 years ago [25,26], but the mechanisms governing death remain unclear. Depletion of T-cells from aged mice significantly delayed death, strongly suggesting that dysregulation of T-cell responses drives mortality in old mice during the acute phase of infection. We found that enhanced thymic function or restoration of thymic size provided benefit for survival to *T. gondii* infection. However, MycTg middle-aged mice showed greatly improved outcomes, whereas the benefit to the iMycTg aged-E model was modest. Consistently, the thymus was considerably larger in MycTg middle-aged mice than in iMycTg aged-E mice, with greater numbers of naïve T-cells in spleen and lymph nodes, whereas iMycTg aged-E mice only showed partial restoration of naïve T-cell numbers in spleen. Thus, the degree of restoration of the naïve T-cell population appears to correlate with protection to *T. gondii* infection in these models. In addition to quantitative increases, analysis of T-cells from the periphery of the MycTg middle-aged and iMycTg aged-E mice indicated qualitative distinctions compared to their age-matched controls. Transcriptomic analysis revealed a more “transcriptionally youthful” gene expression profile of T-cells isolated from iMycTg aged-E hosts compared to WT aged in the steady state. Moreover, T-cells isolated from MycTg middle-aged and iMcyTg aged-E hosts post-infection displayed gene signatures more comparable to WT young than the aging controls. Most notably, an age-associated loss of a Th1 gene signature in the activated CD4 cluster was observed between the WT young and WT aging samples, which was revived in T-cells isolated from mice with restored thymic function. This may be one explanation for the enhanced T-cell-mediated protection against *T. gondii* in MycTg middle-aged and iMycTg aged-E mice, as this parasitic infection directs a Th1-type response [62]. However, alterations to the Th1 response alone are an inadequate explanation as aged mice depleted of CD4 and CD8 T-cells had prolonged survival following *T. gondii* infection, suggesting it is not just the absence of protective responses but the presence of pathological responses that mediate rapid death in aging mice.

Another difference observed between young and old mice was within the Treg subset, with aged mice presenting an overall increase in Treg frequencies in the periphery but with diminishing levels of CD25^+^ Treg, consistent with a previous report [69]. Loss of protective CD25^+^ Treg with age was prevented in the MycTg middle-aged mice but not in iMycTg aged-E mice. This could be a consequence of the differing thymic growth rates between the models and the effects of preventing thymic involution compared to reversal on peripheral T-cell pools. These quantitative and temporal distinctions could help explain the disparity in survival to *T. gondii* infection between MycTg middle-aged and iMycTg aged-E mice. Interestingly, antibody depletion of CD25-expressing Treg [39] in WT middle-aged mice resulted in dramatically increased mortality rates, confirming their protective role during this challenge. It is well-documented that aging drives chronic inflammation [70,71], accompanied by expansion of conventional effector T-cells in the periphery. This could help explain why Treg depletion did not result in *T. gondii* lethality in WT young mice. We suggest that middle-aged mice may have a greater need for CD25^+^ protective Treg to suppress the expanded population of effector subsets and dampen inflammation occurring with aging. Thus, we speculate that the maintenance of CD25^+^ Treg contributes to the striking effectiveness of thymic hyperplasia in preventing lethality to *T. gondii* in middle-aged mice. Additional work is needed to confirm if this, or other mechanisms, underlie the results.

Moderate restoration of thymic cellularity using pharmacological approaches did not protect against viral infection in aging hosts. This susceptibility of aging mice was associated with deterioration of secondary lymphoid organs that seemed unable to support the maintenance of newly generated T-cells [19]. Complementary to these findings, the restoration of thymic size in aged mice increased frequencies of naïve T-cells in the spleen of iMycTg aged-E mice but did not increase naïve T-cells in iLN. The enlarged thymus and the continued export of T-cells into the periphery of the MycTg middle-aged model, however, did prevent the age-associated decline in secondary lymphoid tissue, with mice presenting with a significant increase in naïve T-cells in the iLN. Sonar and colleagues observed heterogeneity in the level of LN atrophy in elderly mice, with some LN displaying much more severe atrophy than others, which consequently affected their ability to retain recent thymic emigrants [72]. We did not conduct extensive examinations of the atrophy of multiple lymphoid organs in our investigations; however, our results are suggestive that thymic involution is a contributing factor to the irreversible LN degeneration observed in aging, and prevention of thymic involution could be protective for lymphoid organ integrity.

We demonstrated that both constitutive and inducible transgenic Myc expression in TEC under the control of the *Psmb11* gene (encoding β5t) promoter can drive the expansion of thymus size. As bipotent TEC progenitors have been demonstrated to undergo a β5t expressing stage during fetal development [30], the observed expansion of both cTEC and mTEC subsets in the constitutive Myc model was not unexpected. However, a β5t lineage trace mouse model revealed that only a small proportion (<5%) of mTEC are derived from β5t-expressing TEC in the adult thymus [44]. Thus, it was unexpected that inducible transgenic Myc expression in aged TEC could drive the expansion of both cTEC and mTEC. However, inducible Myc expression in aged TEC did not consistently drive thymic growth. The Myc transgene may need to be expressed in a rare bipotent TEC progenitor population to successfully drive thymic growth, which may not always occur during the short 2-week induction period that we applied. However, the hCD2 reporter consistently labeled a large fraction of all TEC subsets in the enlarged and unenlarged iMycTg thymi, indicating successful transgene induction, regardless of subsequent growth. The mTEC^hi^ subset of iMycTg aged-E TEC displayed the lowest levels of hCD2, which may suggest that forced Myc expression may inhibit differentiation of mTEC progenitors into mTEC^hi^ subsets. The large frequency of hCD2-labeled mTEC is unexpected when considering the very few mTEC labeled in the β5t lineage trace mouse model induced during postnatal life [44] and warrants further investigation. As β5t is an Aire-dependent tissue-restricted antigen [73], one interesting possibility is that inducibleβ5t expression facilitates Myc induction in a small proportion of Aire^+^ mTEC, transcribing β5t at the time of doxycycline administration, and this population mediates mTEC expansion and thymic growth. Understanding why growth does not always occur in the aged TEC, and which TEC subsets need to express Myc to drive thymic regeneration would offer greater insight into strategies of TEC-mediated thymic rejuvenation.

Another consideration of this thymic regeneration model is the properties of the TEC subsets restored within the aging thymus. Previous work has demonstrated that castration in aging male mice can restore TEC numbers but does not reverse their molecular characteristics, with age-associated changes persisting in the regrown thymus [74]. Moreover, TEC, particularly cTEC, undergo severe morphological changes with age and are dependent on signals from mTEC during regeneration for their transient remodeling [75]. With the expansion of Ly51^−^ UEA^−^ age associated TEC populations [76] observed in the iMycTg aged-E thymi coupled with a reduction in the mature mTEC^hi^ subset, it is possible that induced Myc expression in aging TEC may result in an expansion in number but may not reverse all age-related changes to the TEC subsets. It may alter the trajectory of TEC to differentiate down this age-associated atypical TEC lineage. This warrants further investigation and is an important consideration for the applications of this thymic regeneration model.

Our findings are supported by recent work that used the administration of RANK-Ligand which also results in thymic regrowth. However, this recent work cannot resolve the contribution of TEC versus effects on other cell types, including dendritic cells, T-cells and macrophages which each express receptors for RANK-Ligand [77]. As Psmb11 is exclusively expressed by TEC, our model rigorously establishes that strategies targeting TEC improve T-cell function in aging.

Thymectomy during adulthood was associated with an elevated risk of all-cause mortality and an increased risk of cancer [78]. This result suggests that although age-related changes to the thymus environment have been initiated, the adult thymus still governs the development of T-cells essential for functional immune responses. This highlights that the adult and aging thymus maintains functional potential to enhance immune fitness with age. The models we describe could be used to assess other age-related immunological challenges. Our findings in mice establish that restoring thymic size in old age provides immune advantage and support the importance of TEC-focused thymic regeneration strategies for enhancing T-cell-mediated immunity in the elderly.

## Materials and methods

### Ethics statement

All animal experiments and procedures followed NIH and US government regulations and policies and were approved by the National Cancer Institute Animal Care and Use Committee (LGI-001 and LICB-035).

### Mice

Myc transgenic mice (Myc-hCD2^stopFL^) were obtained from Jackson Laboratory (#020458). Myc^stopFL^ mice were previously generated [31] by inserting a human Myc cDNA preceded by a floxed stop cassette into the ROSA26 locus. The construction was marked by a signaling-deficient truncated version of human CD2 (hCD2). Myc^stopFL^ mice were crossed to β5t-Cre mice to generate the constitutive Myc transgenic model (MycTg) or to β5t-rtTA x tetO-Cre mice to generate the inducible Myc transgenic model (iMycTg). β5t-Cre mice and β5t-rtTA × tetO-Cre mice were donated by Y. Takahama (NCI) and were previously reported [30,44]. Mice described as young were 2–3 months of age, mice described as middle-aged were 8–12 months, and mice described as aged were 15–18 months, unless otherwise specified. Our aged cohort includes a distinct age range to the Jackson and colleagues, 2017 classification of ‘Old Aged’ between 18–24 months [79]. Both females and males were used interchangeably in experiments. Mice were maintained under specific pathogen-free conditions, and experiments were conducted under the approval of the National Institutes of Health Animal Care and Use Committees.

### Doxycycline treatment

Nine to twelve-month-old WT and iMycTg mice were administered with 2 mg/ml of doxycycline (Sigma-Aldrich) in drinking water containing 5% (w/v) sucrose. Mice received doxycycline water for two weeks and were then used for experiments 3–6 months later.

### Thymus ultrasound

iMycTg aged mice were ultrasound-scanned before infection to assess thymus size. WT aged and WT young mice were also scanned as controls. Mice were anesthetized through the continuous administration of 1–4% isoflurane anesthesia (Forane, Baxter Healthcare Co.) using a nose cone and precision-calibrated vaporizer (Somni Scientific, South Park, PA). After induction of anesthesia, hair was removed from the ventral thorax using a chemical hair remover (Nair Hair Remover Church and Dwight Co.) followed by a neutralizing after-wash solution (Epil-Stop). Ultrasound imaging was conducted using a Vevo Imaging Station 3100 (Visualsonics). Mice were placed supine on the animal platform (37 °C) and secured with transparent hypoallergenic tape (Transpore) applied to the hind and forelimbs. Body temperature was monitored via a rectal thermometer. ECG electrodes monitored the heart rate under all legs (Signal gel, Parker). Prewarmed ultrasound gel (Aquasonic Clear) was spread over the chest wall. An MS550 transducer was positioned at the upper 1/3 of the chest so that only the upper part of the thymus was recorded. The ultrasound probe was placed transversely over the thymus to capture cross-sectional images. The ultrasound settings were: Frequency: 40 MHz; power level: 100%; imaging deep: 6–8 mm; frame rate: 232; dynamic range: 60 dB; display map: C5.

### NP-KLH immunization

Myc middle-aged mice were immunized by IP injection of 50 μg NP-KLH in alum. NP-specific serum antibodies were assessed by ELISA on day 14 after immunization, as described in [80].

### *T. gondii* infection

ME-49 type II strain (ATCC 50840) cysts expressing RFP [37], obtained from chronically infected (8–10 weeks post-infection) WT young mice were utilized for experiments. Following euthanasia, the whole brain was homogenized in 1 ml of PBS (pH 7.2), and 20 µl aliquots were used for cyst counts. Mice were infected with 10 cysts diluted in 250 µl of PBS via oral gavage.

### Thymic epithelial cell isolation

The thymus was mechanically disrupted into RPMI complete media (RPMI, 5% newborn calf serum, and 1× master mix of Pen-Strep, l-glutamine, amino acids, sodium pyruvate, and Hepes). TEC were isolated according to the methods stated in [24].

### T-cell isolation

For the spleen, the tissue was mechanically disrupted in RPMI complete media (RPMI, 5% newborn calf serum, and 1× master mix of Pen-Strep, l-glutamine, amino acids, sodium pyruvate, and Hepes) and subsequently treated with 1× ACK Lysis Buffer (Lonza) for 2 min on ice.

### T-cell depletion

The protocol was standardized in our lab based on a previous report [27]. Mice were administered with 0.2 mg of the rat anti-mouse CD4 (GKl.5mAb, IgGZa) and 0.1 mg of the rat anti-mouse CD8 (2.43 mAb IgG2a) by i.p injection in a final volume of 200 ul. The anti-mouse CD4 was administered seven consecutive days before the infection, whereas the anti-CD8 was administered during three consecutive days before the infection. Additional doses of both antibodies were administered every third day for the experiment.

### Treg depletion

One day before *T. gondii* infection, mice received 400 μg of anti-mouse CD25 (IL2Ra, clone: PC-61.5.3; BioXcell) in 200 µl of PBS via i.p. injection.

### Flow Cytometry

Absolute cell numbers were obtained using an Accuri C6 PLUS flow cytometer (BD). TEC were stained in MACS buffer (PBS, 0.5% FBS, 2 mM EDTA) using the following antibodies: anti-CD45.2 clone C363-16A (BioLegend), anti-EpCAM clone G8.8 (Invitrogen), anti-Ly51 clone 6C3 (BioLegend), anti-UEA1 clone B-1065 (Vector Laboratories), anti-CD80 clone 16-10A1 (BioLegend), anti-MHCII clone M5/114.15.2 (BioLegend), anti-hCD2 clone TS1/8 (BioLegend). T-cells were stained in FACS buffer using the following antibodies: anti-TCRβ clone H57-597 (BioLegend), anti-CD4 clone RM4-5 (BioLegend), anti-CD8 clone (BioLegend), anti-CD62L clone MEL-14 (BioLegend), anti-CD44 clone IM7 (BioLegend), anti-PD1 clone 29F.1A12 (BioLegend), and anti-Foxp3 clone FJK-16s (Thermo Fisher). For the intracellular staining of Foxp3, cells were fixed and permeabilized using the Foxp3/Transcription Factor Fixation/Permeabilization Concentrate and Diluent kit (eBioscience) according to the manufacturer’s instructions. For tetramer binding assessment, T-cell preps were incubated with the TGME49 Class II I-Ab tetramers loaded with the AS-15 peptide [81] (NIH Tetramer Facility) for 1 h before the surface staining. Samples were acquired using an LSR Fortessa flow cytometer (Becton Dickinson) and analyzed using the FlowJo software v10 (Becton Dickinson).

### ScRNA-seq library preparation

The sorted cells were loaded on a 10X Genomics chip at a concentration of 1,200 cells/μl and loaded into the 10× chromium controller for droplet generation. The libraries were generated using the 10× Genomics 3-prime version 3 chemistry and prepared according to the manufacturer’s recommendations. The cDNA libraries underwent three sequencing runs on a NextSeq instrument using a NextSeq 2k P3 100-cycle flowcell.

### scRNA-Seq data analysis

Publicly available scRNAseq fastq files were downloaded from the Broad Institute single cell portal [47] or the synapse database, repository syn22255433 [48]. All data was remapped using CellRanger version 6.0.2. The publicly available and in-house generated data was loaded into R using the Read10X_h5 function from Seurat. Doublet detection was performed with the scDblFinder function, using default settings from the scDblFinder package. Quality metrics were calculated for each cell using the perCellQCMetrics from the scuttle package. Using the number of UMI and number of expressed genes per cell, the isOutlier function from the scuttle package was used to flag low-quality cells that differed 2 median absolute deviations below the threshold for each library; the variable log was set to TRUE. This resulted in 2,000–6,000 high-quality cells at a sequencing depth of 30,000–60,000 reads per cell.

The count data from high-quality cells was loaded into a SeuratObject using the CreateSeuratObject, with min.cells set to 3. The data was normalized and scaled using Seurat’s NormalizeData and ScaleData functions. To overcome differences in cell type composition between the datasets generated by different labs, the datasets were normalized separately, and the top 2,000 highly variable genes (HVG) were selected for each dataset separately using the FindVariableFeatures function from Seurat, leveraging the variance stabilizing transformation (vst) method. A common set of HVGs was selected using the SelectIntegrationFeatures function from Seurat. Principal component analysis (PCA) was performed using the multiBatchPCA function from the batchelor package and batch effects due to differences in infection status, genotype and age were corrected using the reducedMNN function from batchelor. The corrected PCA matrix was used to generate a UMAP using the RunUMAP function from Seurat. A shared nearest neighbor graph was generated using the top 50 PCs from the corrected PCA matrix and clusters were identified using the FindClusters function from Seurat, leveraging the smart local moving algorithm, and the resolution set to 0.25. Clusters containing non-T lineage cells, as identified based on an absence of *Cd3e*, *Trac*, and *Cd7* expression, were removed.

Genes detected in less than three cells were removed from the T lineage cells, and the data was normalized and scaled. The top 2000 HVGs were selected using the FindVariableFeatures function from Seurat using the vst method. A PCA matrix was generated and corrected as previously described. Next, a UMAP was generated and clusters were identified as previously described, albeit with resolution set to 0.5. Subclusters were identified as needed using the FindSubCluster function from Seurat. The obtained clusters were annotated based on marker gene expression. Subsequently, this annotation was used to classify cells that did not cluster in transcriptionally homogenous groups using an elastic net classifier. This was done using the glmnet and cv.glmnet functions from the glmnet package, using a randomly selected subset of 1,000 cells if possible for each annotated population as the training data, and alpha set to 0.1, family set to multinomial and type.measure set to class. Annotations were predicted using the predict function from glmnet, while providing the lambda obtained from the cross-validation. Each cell was assigned an annotation based on the maximal probability obtained.

Regulatory T-cells were isolated based on their annotation, and lowly expressed genes were removed as described previously. The cells were analyzed as described above, except for the resolution being set to 0.3 to identify clusters. These clusters were annotated based on their expression of *Il2ra* and *Pdcd1* as CD25^+^ and PD1^+^, respectively.

The different analyses performed on the various experimental conditions were performed as follows: briefly, the data was normalized and scaled and HVG were selected using the NormalizeData, ScaleData, and FindVariableFeatures from Seurat. Batch correction was done using the multiBatchPCA and reducedMNN functions from the batchelor package. UMAPs were generated as described above.

### Gene score generation

Aging gene scores for all CD4 or CD8 cells (Fig 8G) were obtained as follows: differential gene expression was done between young and old mice, including in house generated and publicly available data [47,48]. For the CD4 T-cells, naïve CD4, EM CD4, and Treg were selected, while for the CD8 T-cells, naïve CD8, EM CD8, and CM CD8 were retained. This was done only using uninfected mice obtained from all applicable datasets. Briefly, the lmFit, contrasts.fit and eBayes functions from the limma package were used to perform the analysis, with age used as contrasts whereas the annotation and dataset were provided as additional variables. The topTable function from limma, specifying the Benjamini-Hochberg method, was used to extract DEG that had a cutoff of <0.05 for the adjusted *p*-values and a log2 fold change ≤−0.25. This resulted in a small set of genes upregulated in aged CD4 or CD8 T-cells, which was then used to calculate gene set scores using the enrichIt function from the escape library, specifying the UCell method.

We generated cell-type-specific aging scores using our in-house generated data (S6A and S6B Fig). To do this, we used the same approach as above, but with notable` differences. We used only our in-house generated data, from which we retained the Naïve CD4, Naïve CD8, Treg, Effector memory CD4, Central memory CD8, iNKT, and Effector memory CD8 cells from the steady state samples. Subsequently, we performed differential gene expression analysis using the previously mentioned approach, and isolated DEG that had an adjusted *p*-value below 0.05, and an absolute log2 fold change ≥0.25. We used these genes to generate aging scores for genes upregulated in aged mice or young mice as specified above.

Finally, we generated infection scores. Here, we used our in-house generated data, from which we retained the naïve CD4, naïve CD8, Treg, EM CD4, CM CD8, iNKT, and EM CD8, from the WT samples. We performed differential gene expression and retained DEG that had an adjusted *p*-value below 0.05 and an absolute log2 fold change ≥0.25. Gene scores were generated using DEG upregulated in WT young infected and WT young steady state samples.

### ScRNA-Seq data visualization

Heatmaps were generated using pseudobulk data. This was done by summing the gene expression counts for each gene in the different populations using the AggregateExpression function from Seurat. Next, a DESeq object was generated, size factors were calculated, and normalized counts, using the DESeqDataSetFromMatrix and estimateSizeFactors functions from the DESeq2 package. The normalized data were log-transformed using the log1p function from Seurat, using 1 as a pseudo count. The normalized and log-transformed data were scaled row-wise using the scale_minmax function from dynutils and visualized using the pheatmap function from the pheatmap package. Density plots were generated using the kde2d function from the MASS package, and each cell was provided a specific value using the findInterval function. Each density estimate for the different conditions was scaled between 0 and 1. The data were visualized using ggplot2. Stacked bar charts were generated by calculating the percentage for each condition and visualized using ggplot2 and ggalluvial. Scatterplots were generated using ggplot2, whereas dot plots were generated using the DotPlot function from Seurat.

### Bulk T-cell receptor β sequencing data

Total CD8 T-cells (TCRβ^+^, CD8^+^), naïve CD8 T-cells (TCRβ^+^, CD8^+^, CD62L^+^ CD44^−^), or memory CD8 T-cells (TCRβ^+^, CD8^+^, CD62L^−^ CD44^+^) were cell sorted and snap frozen. The gDNA was then extracted from the samples using the Qiagen DNeasy Blood and Tissue Kit and mini spin columns according to the manufacturer’s instructions. The samples were then ship to Adaptive Biotech for TCRβ chain sequencing. Diversity metrics were obtained from the immunoSEQ Analyzer platform provided by Adaptive Biotech. Bar charts were generated using ggplot2.

### Cytokine measurement

*T. gondii*-infected mice were euthanized on day 9 after parasite inoculation. Blood samples were collected and allowed to clot at room temperature for 30 min to 1 hour. Samples were then centrifuged at 1,000*g* for 10 min at 4 °C. The supernatant (serum) was collected into a fresh tube and immediately frozen at −80 °C. Serum samples were sent to Eve Technologies (Calgary, AB) for cytokine quantification using the Mouse Cytokine 32-Plex Discovery Assay.

### H & E staining and sectioning

Thymi were collected from 9-week WT mice or 6-month WT and MycTg mice. Thymus samples were fixed in 10% neutral buffered formalin (Thermo Scientific), embedded in paraffin wax, sectioned at a thickness of 8 µm, and stained with hematoxylin and eosin (H&E). Staining was performed by Histoserv, . In briefly, the slides were treated with xylene, then hydrated through graded alcohols up to water. Slides were then stained with Carazzi’s hematoxylin, washed in water, and placed in 95% ethanol. Slides were then counterstained with eosin-phloxine, dehydrated through graded alcohols, cleared in xylene, and coverslipped using permount as mounting media. Central sections were imaged using a Carl Zeiss Axioscan.Z1 microscope with a Plan-Apochromat 20×/0.8 NA objective and a Hitachi HV-F203 CCD camera with a 4.4 mm pixel size. Acquisition parameters included a 3ms flash duration with an LED set to 152% intensity.

### Statistics

GraphPad Prism v10 for macOS was used to construct graphs and analyze data. Differences between two-groups were determined using a paired or unpaired parametric *t* test or non-parametric Mann–Whitney test according to the data distribution. For multigroup analysis, we performed an ANOVA test followed by Tukey’s multiple comparison test for data with a normal distribution or a Kruskal–Wallis test followed by Dunn’s multiple comparison test for data with a non-normal distribution. Survival analyses were performed using the Kaplan–Meier method. The coefficient of correlation (*R*²) was used to evaluate the linearity between thymus cellularity and the volume or area of the thymus as predicted by ultrasound scanning. A *p*-value of less than 0.05 was considered statistically significant for all tests. Significance levels are indicated as follows: *p < 0.05, **p < 0.01, ***p < 0.001, and ****p < 0.0001.

## Supporting information

S1 Fig Enforced expression of Myc in TEC. (**A**) Representative FACS histograms showing the staining of hCD2 (Myc reporter, see schematic in Fig 1A and Materials and methods) in CD45^+^ cells and the indicated thymic epithelial cell (TEC) subsets from a WT middle-aged (8–12 months of age) and a representative MycTg middle-aged mice (left). Bar plot showing the percentage of hCD2^+^ cells in CD45^+^ cells and the indicated TEC subsets (right). *N* = 3 mice from a representative experiment. The data underlying this figure can be found in S7 File.
(TIFF)

S2 Fig Myc overexpression in TEC prevents the age-associated decline in naïve CD4 and CD8 T-cells in the inguinal lymph nodes. (**A**) Total cellularity of the two merged inguinal lymph nodes (iLN) in WT young (2–3 months of age), WT middle-aged (8–12 months of age) and MycTg middle-aged (8–12 months of age) mice. (**B**) Representative FACS plots of the expression of TCRβ in the iLN live cells. Bar plots showing the percentages and absolute numbers of TCRβ^+^ cells in the indicated mouse groups. (**C**) FACS plots and bar plots of the CD4 and CD8 T-cells (pre-gated in TCRβ^+^ cells) frequencies and absolute numbers. (**D**) CD62L and CD44 expression in CD4 (top) and CD8 (bottom) T-cells. Bar plots depicting percentages and quantitation of CD62L^+^ CD44^−^ (naive), CD62L^+^ CD44^+^ central memory (CM) and CD62L^−^ CD44^+^ effector memory (EM). (**E**) FACS plots showing Foxp3 staining in CD4 T-cells. Bar graphs show the percentages and absolute numbers of Foxp3^+^ Treg cells. *p < 0.05, **p < 0.01, ***p < 0.001, and ****p < 0.0001. The data underlying this figure can be found in S7 File.
(TIFF)

S3 Fig Identification of CD25+ Foxp3+ Treg in the spleen after αCD25 administration. (**A**) Mice were IP-injected with αCD25-depleting antibody or vehicle (refer to Fig 3C and Materials and methods) the day before *Toxoplasma gondii* infection and euthanized on day 9 post-infection for Treg cells staining. FACS plots showing the Foxp3 and CD25 staining on TCRβ^+^ CD4 T-cells in the spleen. Bar plots show the percentages and numbers of total Foxp3^+^ and CD25^+^ Foxp3^+^ Treg cells. The data underlying this figure can be found in S7 File.
(TIFF)

S4 Fig A subset of iMycTg mice did not show thymic regrowth. (**A**) Bar plot showing the percentage of hCD2^+^ cells in CD45^+^ cells and the indicated thymic epithelial cells (TEC) subsets from WT Aged, iMycTg Aged-U (unenlarged) (15–18 months of age) and iMycTg Aged-E (enlarged) (15–18 months of age) thymi. Mice were analyzed on average 4.5 months after induction (range: 3.8–5.1 months). (**B**) Mice were infected with 10 cysts of *Toxoplasma gondii*. Thymus cellularity was assessed on days 0, 3, and 9 after inoculation. (**C**) Representative ultrasound images of unenlarged and enlarged thymi from iMycTg mice at steady state. Mice were analyzed 4.7 months after induction. (**D**) The correlation between ultrasound imaging-based thymus volume (Vol.) or area versus thymic cellularity. *p < 0.05 and ****p < 0.0001. The data underlying this figure can be found in S7 File.
(TIFF)

S5 Fig Restored thymic function did not alter naïve T-cell populations in the inguinal lymph node. (**A**) Total cellularity of the two merged inguinal lymph nodes (iLN) in WT young (2–3 months of age), WT aged (15–18 months of age) and iMycTg aged-E (enlarged) (15–18 months of age) mice. (**B**) Representative FACS plots of the expression of TCRβ in the iLN live cells. Bar plots showing the percentages and absolute numbers of TCRβ^+^ cells. (**C**) Representative FACS plots and bar plots of the CD4 and CD8 T-cell frequencies and absolute numbers (pre-gated on TCRβ^+^ cells). (**D**) CD62L and CD44 expression in TCRβ^+^ CD4 (top) and TCRβ^+^ CD8 (bottom) T-cells. Bar plots depicting percentages and quantitation of CD62L^+^ CD44^−^ (naive), CD62L^+^ CD44^+^ central memory (CM) and CD62L^−^ CD44^+^ effector memory (EM) in the indicated mice groups. (**E**) FACS plots showing the Foxp3 staining in TCRβ^+^ CD4 T-cells. Bar graphs show the percentages and absolute numbers of Foxp3^+^ Treg cells. Mice were analyzed 6 months after induction. *p < 0.05, **p < 0.01. The data underlying this figure can be found in S7 File.
(TIFF)

S6 Fig Restoring thymic function partially reverts the aging score of different T-cell clusters. Scaled heatmaps visualizing aging scores based on **(A)** genes upregulated or **(B)** downregulated in WT young (2–3 months of age) versus WT Aged (15–18 months of age) mice (see Materials and methods), per indicated T-cell cluster from WT Young, WT Aged or iMycTg Aged-E steady state samples described in Fig 8B. The data underlying this figure can be found in S7 File.
(TIFF)

S7 Fig Restoring thymic function did not alter parasite-restricted CD4 T-cells following infection. Representative FACS plots showing frequencies of parasite-specific (I-A(b)-restricted) TCRβ^+^ CD44^+^ CD4 T-cells at (**A**) day 7 or (**B**) day 9 after *Toxoplasma gondii* infection in the indicated mouse groups. Mice described as young were 2–3 months of age, mice described as middle-aged were 8–12 months, and mice described as aged were 15–18 months. Bar plots show parasite-specific TCRβ^+^ CD44^+^ CD4 T-cell frequencies and numbers. The iMyc mice were analyzed 3.6 months after induction. **p < 0.01 and ***p < 0.001. The data underlying this figure can be found in S7 File.
(TIFF)

S8 Fig Restoring thymic function did not alter serum cytokines following infection. (**A**) WT young (2–3 months of age), WT aged (15–18 months of age) and iMycTg aged-E (15–18 months of age) mice were infected with 10 cysts of *Toxoplasma gondii* and serum was collected after euthanasia on day 9 post-infection for cytokine measurement (Eve Technologies, Calgary, AB, see Materials and methods). Serum samples from uninfected WT young mice were used as an additional control. iMycTg mice were analyzed on average 4.2 months after induction (range: 3.7–5.1 months). *p < 0.05, **p < 0.01 and ***p < 0.001. The data underlying this figure can be found in S7 File.
(TIFF)

S9 Fig Flow analyses of splenic T-cells after *Toxoplasma gondii* infection. Representative FACS plots showing CD62L and CD44 expression in (**A**) TCRβ^+^ CD4 T-cells and (**B**) TCRβ^+^ CD8 T-cells in WT young (2–3 months of age), WT aged (15–18 months of age) and iMycTg aged-E (enlarged) (15–18 months of age) mice, 7 days post *T. gondii* infection. Bar plots depict percentages and numbers of CD62L^+^ CD44^−^ (naive), CD62L^+^ CD44^+^ central memory (CM) and CD62L^−^ CD44^+^ effector memory (EM) in the indicated mouse groups. Mice were infected 3.6 months after induction. *p < 0.05, **p < 0.01, and ***p < 0.001. The data underlying this figure can be found in S7 File.
(TIFF)

S10 Fig Transcriptome analysis of T-cells isolated from mice infected with *Toxoplasma gondii.* Mice were infected with *T. gondii* and sacrificed on day 12 post-infection for scRNA-Seq analysis on splenic T-cells (refer to Fig 9). (**A**) A scaled heatmap showing the expression of genes previously identified as being associated with the Th1 response [63]. (**B**) and (**C**) Scaled heatmaps displaying infection scores generated using genes (**B**) upregulated or (**C**) downregulated in infected WT young samples compared to WT young (2–3 months of age) steady state samples per indicated T-cell cluster, per individual mouse group listed. The data underlying this figure can be found in S7 File.
(TIFF)

S1 File Differentially expressed genes upregulated or downregulated in WT aged T-cell clusters compared to WT young T-cell clusters. Gene lists of genes upregulated ‘Up in old samples’ or downregulated ‘Up in young samples’ in WT old T-cell clusters compared to WT young T-cell clusters. Each tab is an individual T-cell cluster, as labeled.
(XLSX)

S2 File Biological processes associated with genes upregulated in WT aged T-cells, compared to WT young T-cells, per cluster. Each tab is an individual T-cell cluster, as labeled.
(XLSX)

S3 File Biological processes associated with genes downregulated in WT aged T-cells, compared to WT young T-cells, per cluster. Each tab is an individual T-cell cluster, as labeled.
(XLSX)

S4 File Differentially expressed genes upregulated or downregulated in WT young infected T-cell clusters compared to WT young uninfected T-cell clusters. Gene lists of genes upregulated ‘Up in infected samples’ or downregulated ‘Up in uninfected samples’ in WT young infected T-cell clusters compared to WT young uninfected T-cell clusters. Each tab is an individual T-cell cluster, as labeled.
(XLSX)

S5 File Biological processes associated with genes upregulated in WT young infected T-cells, compared to WT young uninfected T-cells, per cluster. Each tab is an individual T-cell cluster, as labeled.
(XLSX)

S6 File Biological processes associated with genes downregulated in WT young infected T-cells, compared to WT young uninfected T-cells, per cluster. Each tab is an individual T-cell cluster, as labeled.
(XLSX)

S7 File The numerical values underlying each figure. Each tab contains the raw data used to generate an individual panel of the indicated figure number.
(XLSX)

## Abbreviations

CM: central memory
DEG: differentially expressed genes
EM: effector memory
HVG: highly variable genes
IL-7: interleukin-7
iLN: inguinal lymph nodes
iMycTg: inducible Myc transgenic model
KGF: keratinocyte growth factor
MFI: median fluorescence intensity
MycTg: Myc transgenic model
NP-KLH: nitrophenyl acetyl conjugated to keyhole limpet hemocyanin
PCA: principal component analysis
scRNA-Seq: single-cell RNA sequencing
TEC: thymic epithelial cells
WT: wild type
