# Supplementary figures and images for: Enhancing thymic function improves T-cell reconstitution and immune responses in aged mice
Source: PLoS Biol. 2025 Jul 28;23(7):e3003283. doi: 10.1371/journal.pbio.3003283 (PMC12303306; doi:10.1371/journal.pbio.3003283)

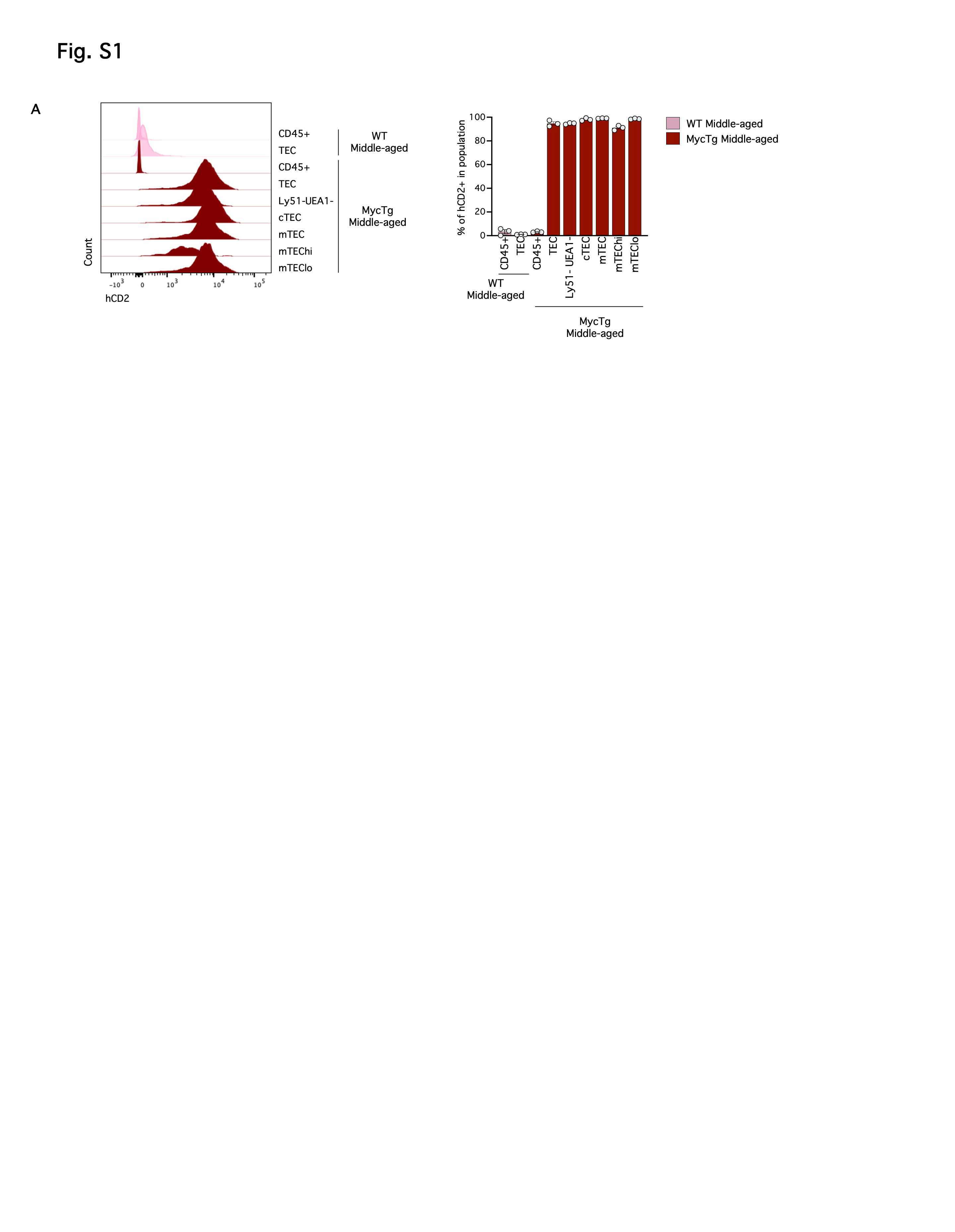

Supplement: S1 Fig — (A) Representative FACS histograms showing the staining of hCD2 (Myc reporter, see schematic in Fig 1A and Materials and methods) in CD45+ cells and the indicated thymic epithelial cell (TEC) subsets from a WT middle-aged (8–12 months of age) and a representative MycTg middle-aged mice (left). Bar plot showing the percentage of hCD2+ cells in CD45+ cells and the indicated TEC subsets (right). N = 3 mice from a representative experiment. The data underlying this figure can be found in S7 File. (TIFF) [file pbio.3003283.s001.tiff]

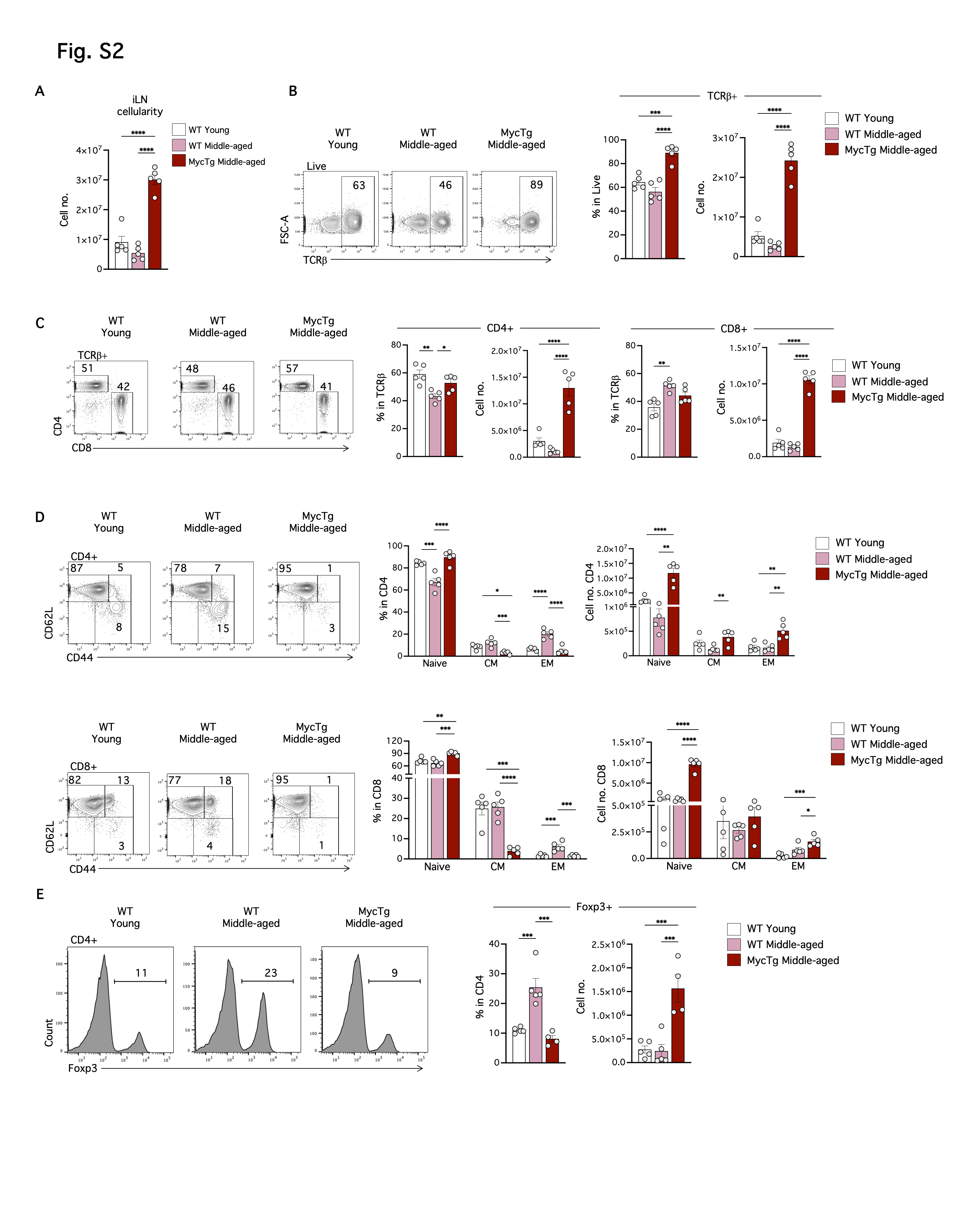

Supplement: S2 Fig — (A) Total cellularity of the two merged inguinal lymph nodes (iLN) in WT young (2–3 months of age), WT middle-aged (8–12 months of age) and MycTg middle-aged (8–12 months of age) mice. (B) Representative FACS plots of the expression of TCRβ in the iLN live cells. Bar plots showing the percentages and absolute numbers of TCRβ+ cells in the indicated mouse groups. (C) FACS plots and bar plots of the CD4 and CD8 T-cells (pre-gated in TCRβ+ cells) frequencies and absolute numbers. (D) CD62L and CD44 expression in CD4 (top) and CD8 (bottom) T-cells. Bar plots depicting percentages and quantitation of CD62L+ CD44− (naive), CD62L+ CD44+ central memory (CM) and CD62L− CD44+ effector memory (EM). (E) FACS plots showing Foxp3 staining in CD4 T-cells. Bar graphs show the percentages and absolute numbers of Foxp3+ Treg cells. *p < 0.05, **p < 0.01, ***p < 0.001, and ****p < 0.0001. The data underlying this figure can be found in S7 File. (TIFF) [file pbio.3003283.s002.tiff]

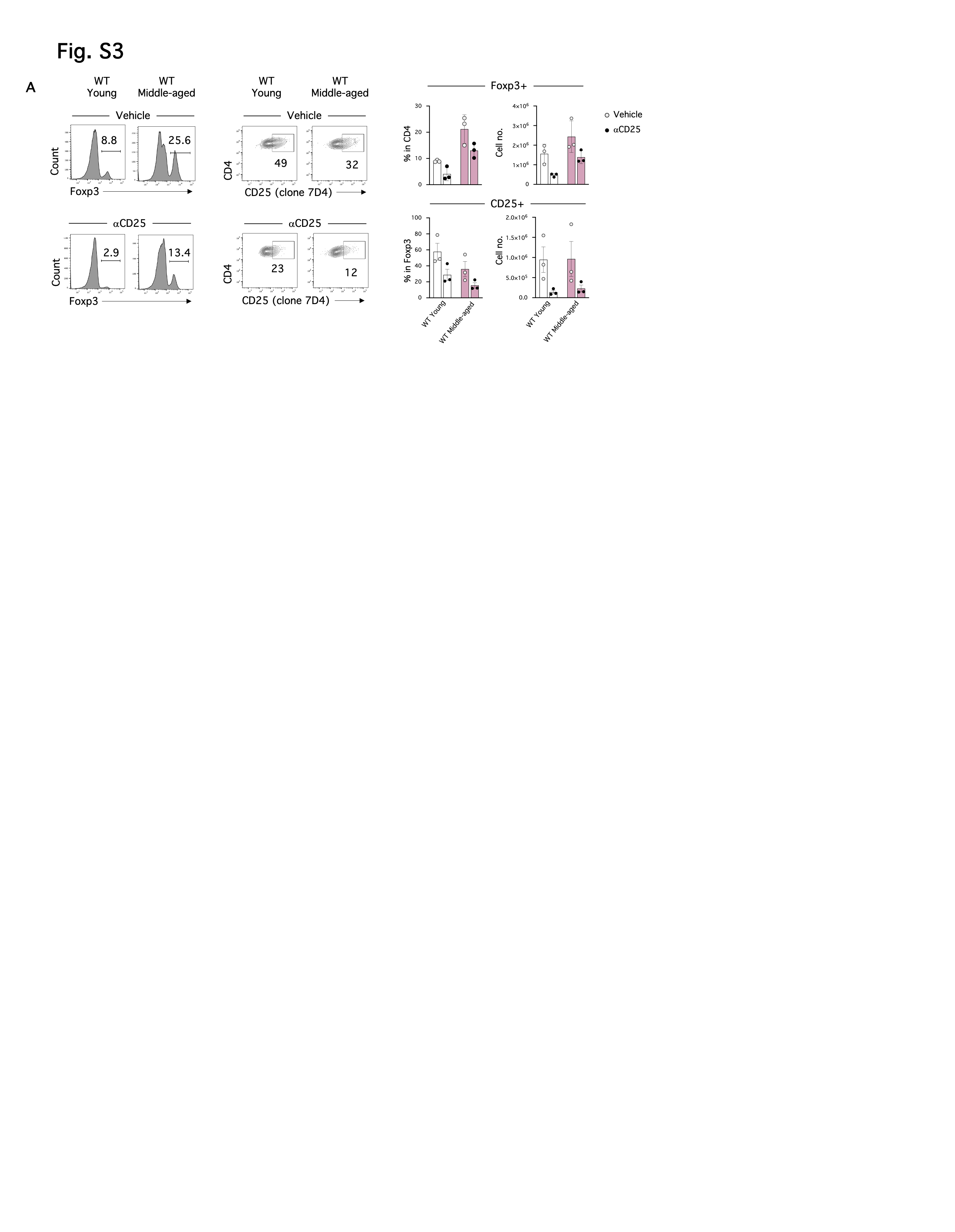

Supplement: S3 Fig — (A) Mice were IP-injected with αCD25-depleting antibody or vehicle (refer to Fig 3C and Materials and methods) the day before Toxoplasma gondii infection and euthanized on day 9 post-infection for Treg cells staining. FACS plots showing the Foxp3 and CD25 staining on TCRβ+ CD4 T-cells in the spleen. Bar plots show the percentages and numbers of total Foxp3+ and CD25+ Foxp3+ Treg cells. The data underlying this figure can be found in S7 File. (TIFF) [file pbio.3003283.s003.tiff]

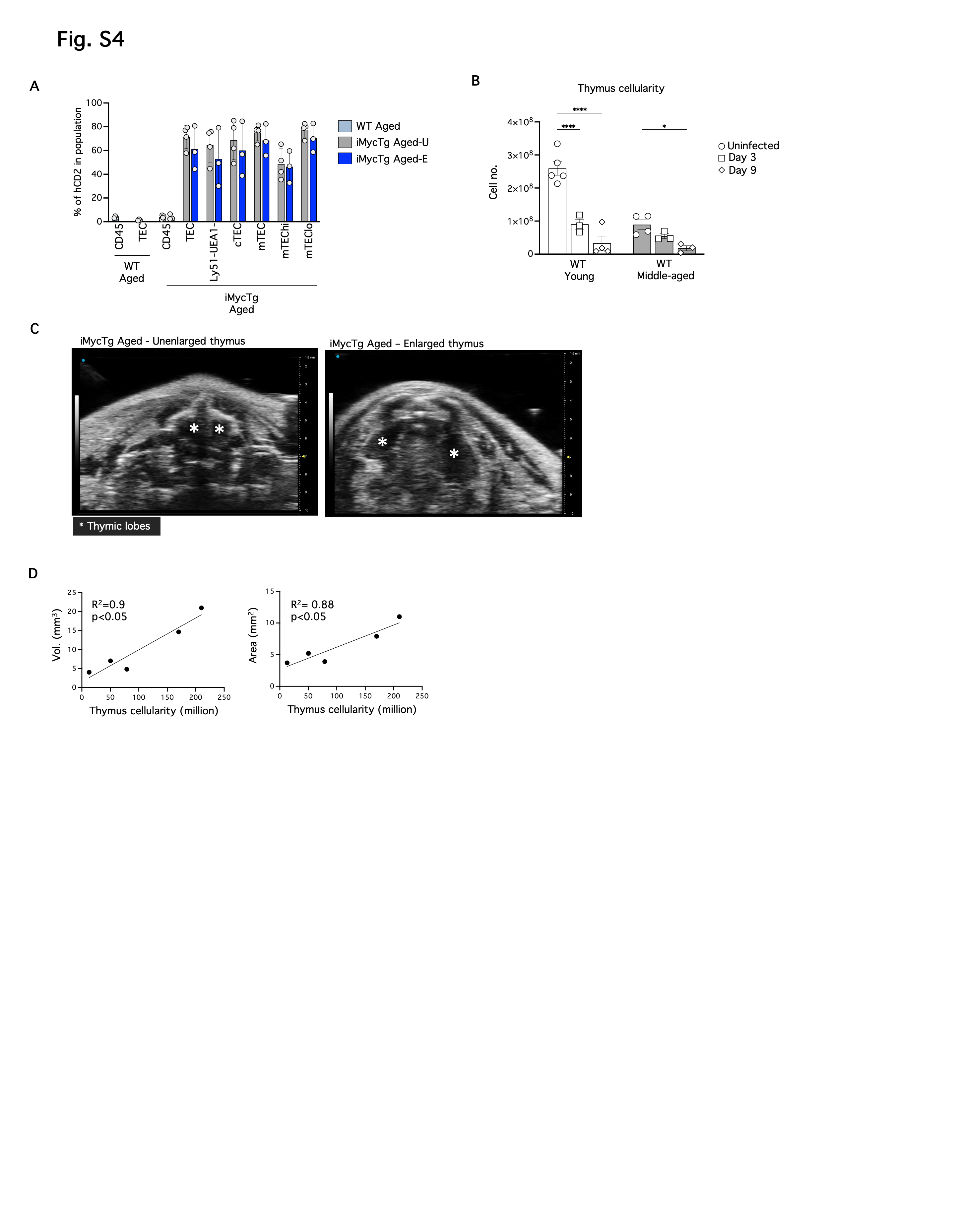

Supplement: S4 Fig — (A) Bar plot showing the percentage of hCD2+ cells in CD45+ cells and the indicated thymic epithelial cells (TEC) subsets from WT Aged, iMycTg Aged-U (unenlarged) (15–18 months of age) and iMycTg Aged-E (enlarged) (15–18 months of age) thymi. Mice were analyzed on average 4.5 months after induction (range: 3.8–5.1 months). (B) Mice were infected with 10 cysts of Toxoplasma gondii. Thymus cellularity was assessed on days 0, 3, and 9 after inoculation. (C) Representative ultrasound images of unenlarged and enlarged thymi from iMycTg mice at steady state. Mice were analyzed 4.7 months after induction. (D) The correlation between ultrasound imaging-based thymus volume (Vol.) or area versus thymic cellularity. *p < 0.05 and ****p < 0.0001. The data underlying this figure can be found in S7 File. (TIFF) [file pbio.3003283.s004.tiff]

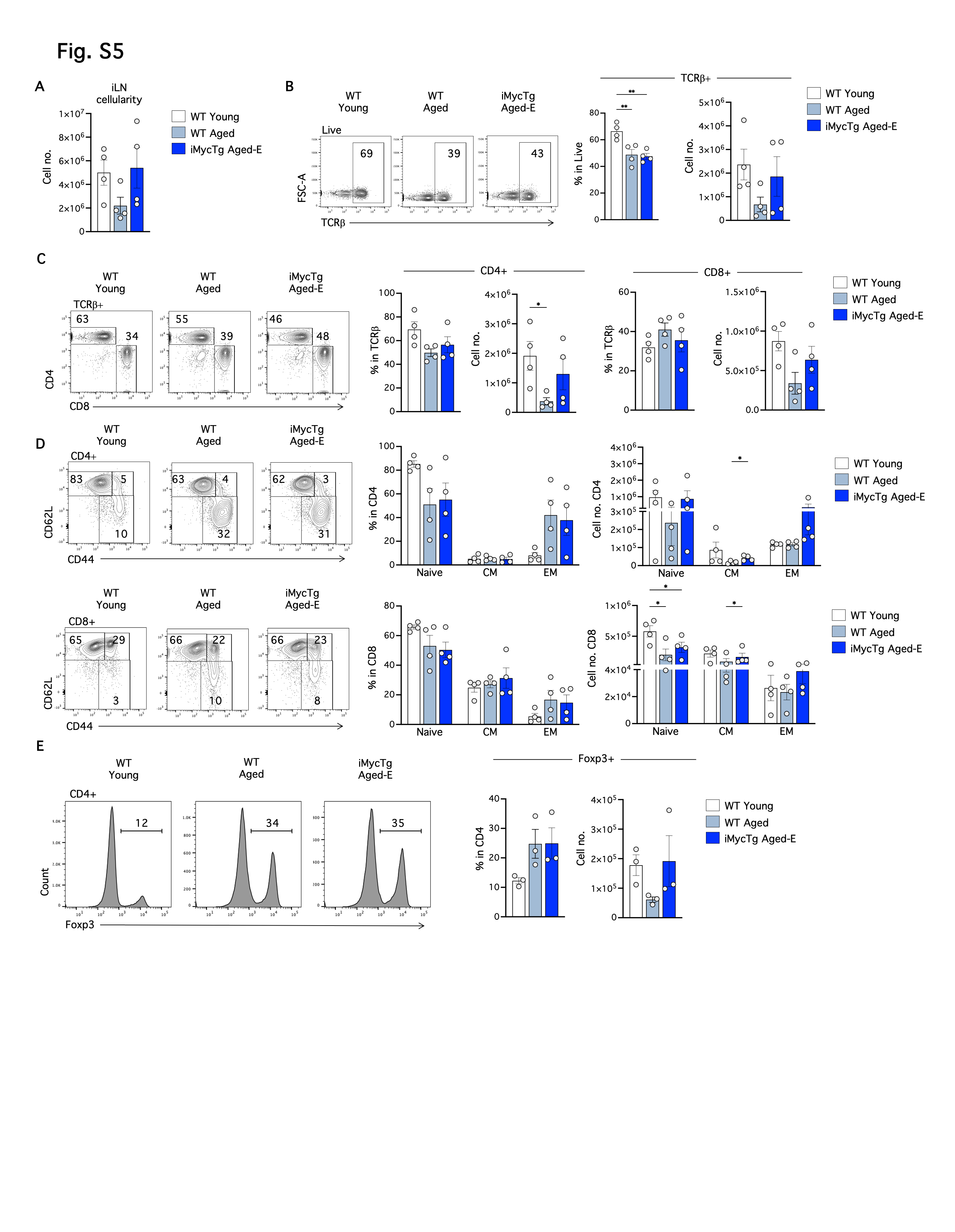

Supplement: S5 Fig — (A) Total cellularity of the two merged inguinal lymph nodes (iLN) in WT young (2–3 months of age), WT aged (15–18 months of age) and iMycTg aged-E (enlarged) (15–18 months of age) mice. (B) Representative FACS plots of the expression of TCRβ in the iLN live cells. Bar plots showing the percentages and absolute numbers of TCRβ+ cells. (C) Representative FACS plots and bar plots of the CD4 and CD8 T-cell frequencies and absolute numbers (pre-gated on TCRβ+ cells). (D) CD62L and CD44 expression in TCRβ+ CD4 (top) and TCRβ+ CD8 (bottom) T-cells. Bar plots depicting percentages and quantitation of CD62L+ CD44− (naive), CD62L+ CD44+ central memory (CM) and CD62L− CD44+ effector memory (EM) in the indicated mice groups. (E) FACS plots showing the Foxp3 staining in TCRβ+ CD4 T-cells. Bar graphs show the percentages and absolute numbers of Foxp3+ Treg cells. Mice were analyzed 6 months after induction. *p < 0.05, **p < 0.01. The data underlying this figure can be found in S7 File. (TIFF) [file pbio.3003283.s005.tiff]

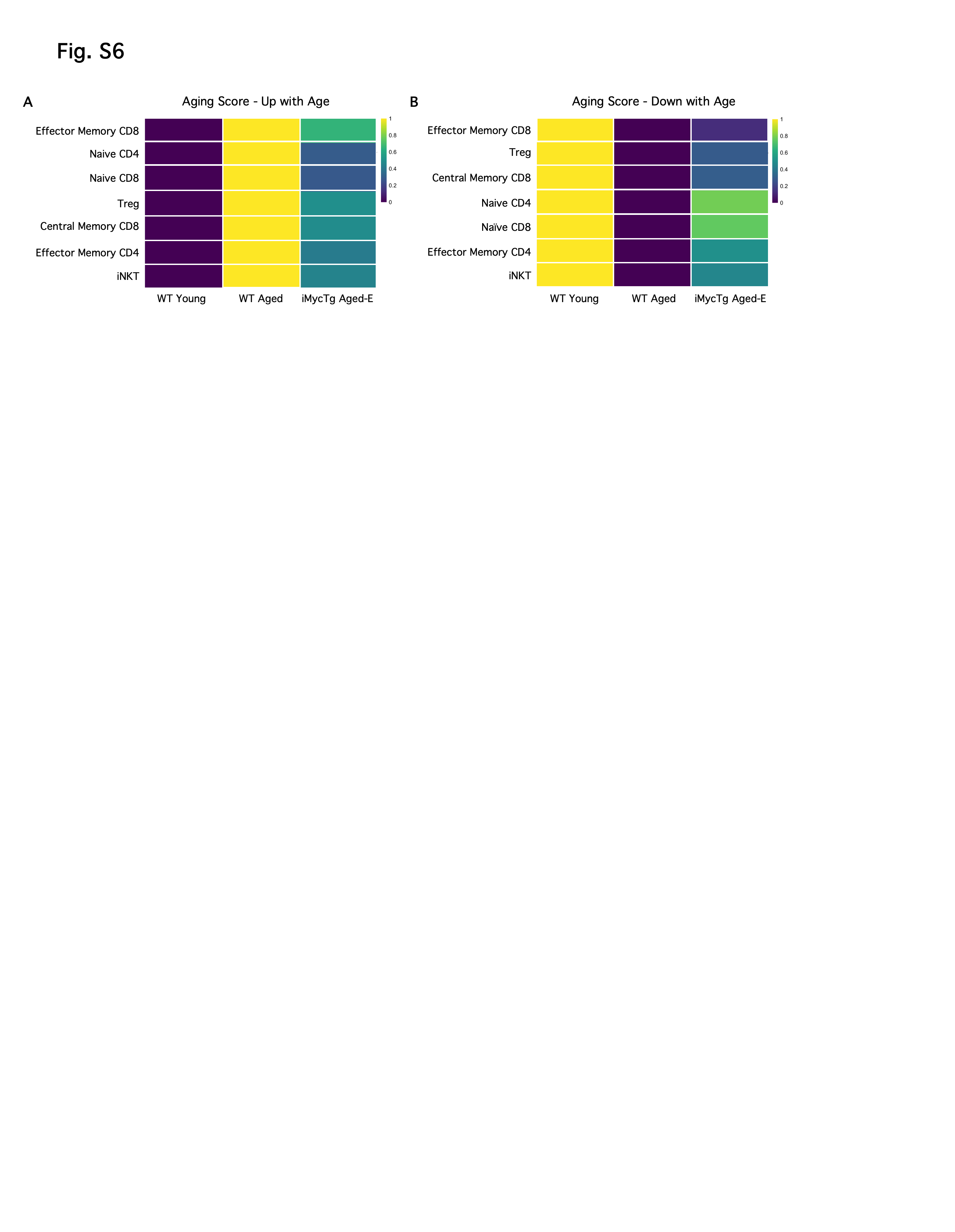

Supplement: S6 Fig — Scaled heatmaps visualizing aging scores based on (A) genes upregulated or (B) downregulated in WT young (2–3 months of age) versus WT Aged (15–18 months of age) mice (see Materials and methods), per indicated T-cell cluster from WT Young, WT Aged or iMycTg Aged-E steady state samples described in Fig 8B. The data underlying this figure can be found in S7 File. (TIFF) [file pbio.3003283.s006.tiff]

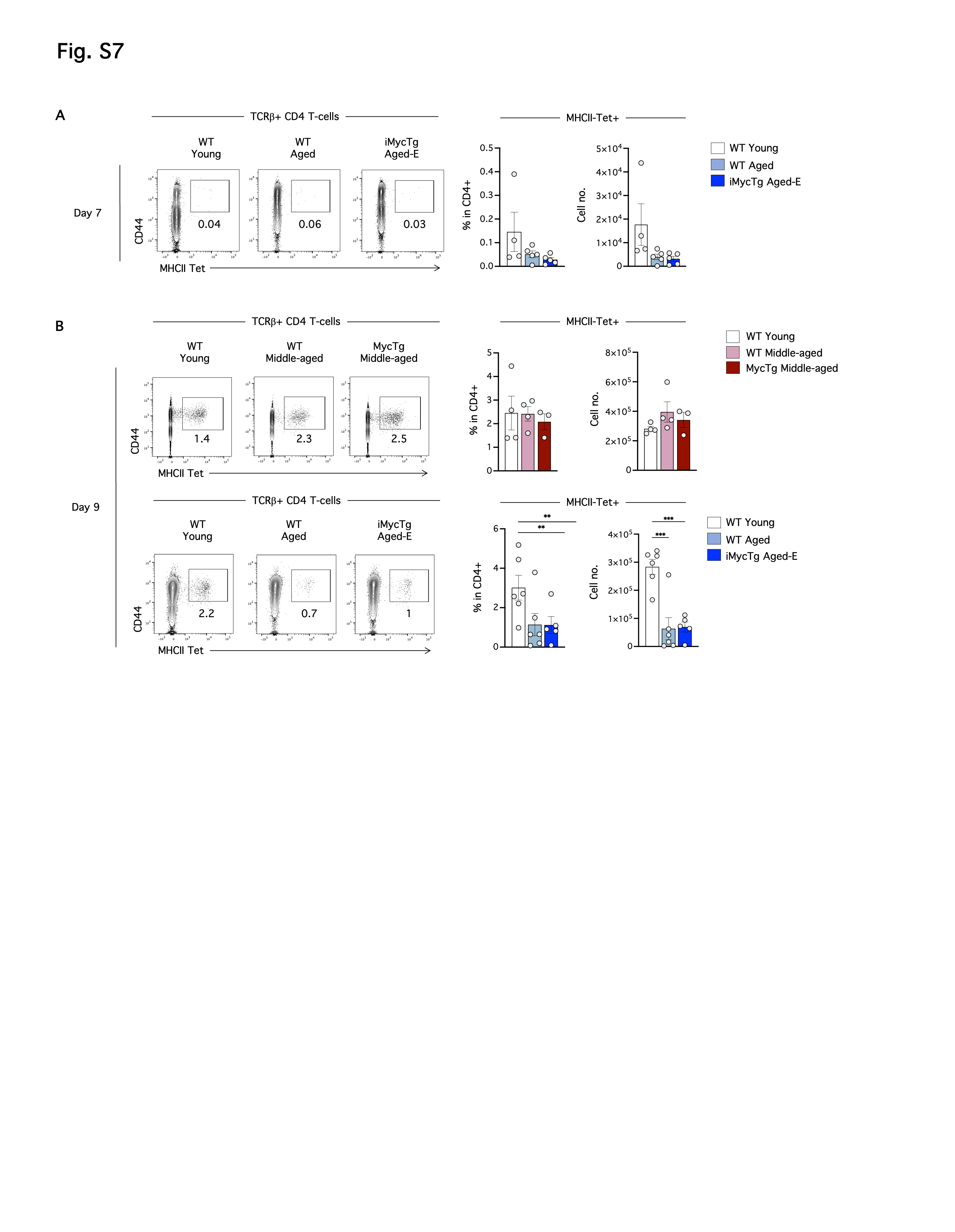

Supplement: S7 Fig — Representative FACS plots showing frequencies of parasite-specific (I-A(b)-restricted) TCRβ+ CD44+ CD4 T-cells at (A) day 7 or (B) day 9 after Toxoplasma gondii infection in the indicated mouse groups. Mice described as young were 2–3 months of age, mice described as middle-aged were 8–12 months, and mice described as aged were 15–18 months. Bar plots show parasite-specific TCRβ+ CD44+ CD4 T-cell frequencies and numbers. The iMyc mice were analyzed 3.6 months after induction. **p < 0.01 and ***p < 0.001. The data underlying this figure can be found in S7 File. (TIFF) [file pbio.3003283.s007.tiff]

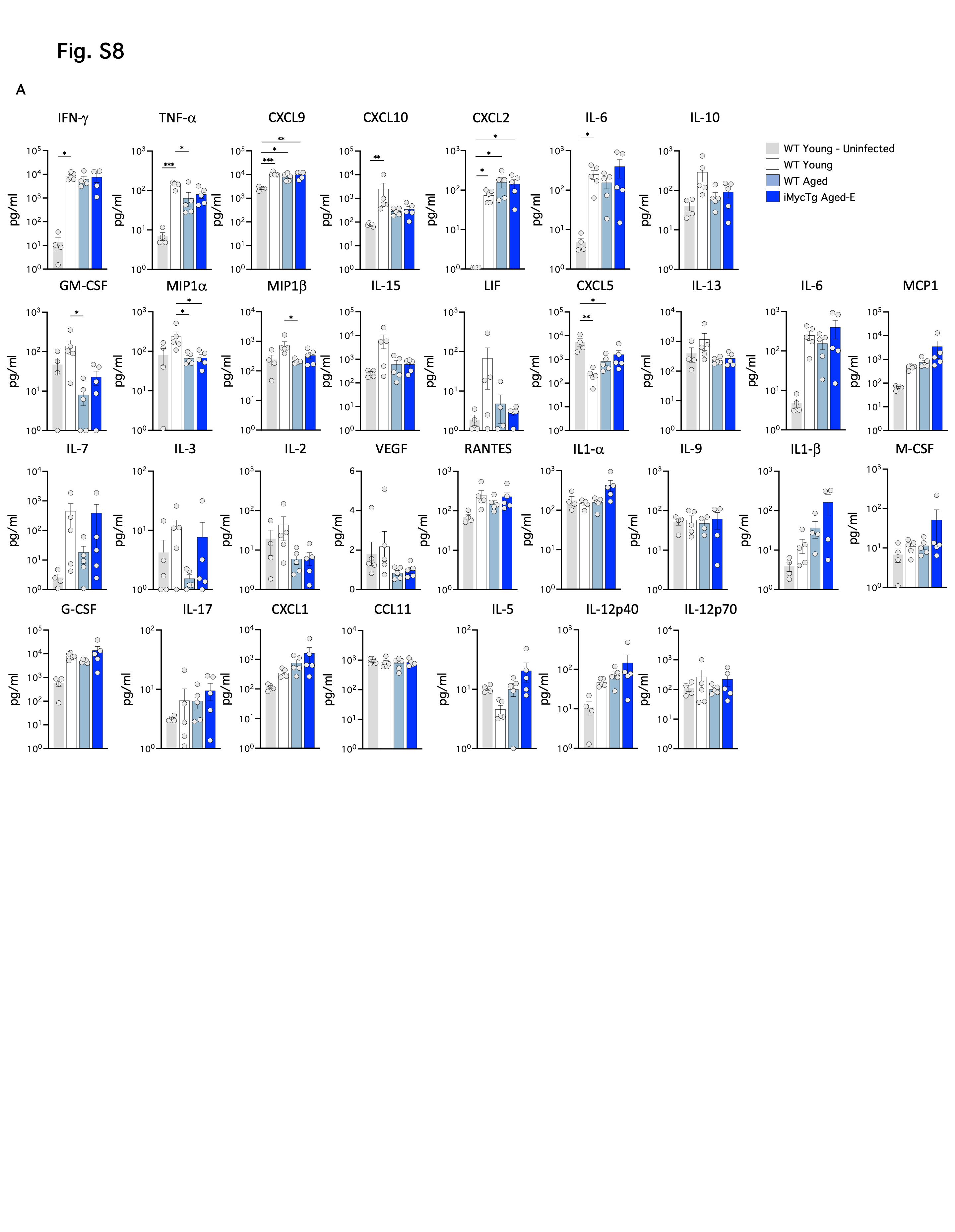

Supplement: S8 Fig — (A) WT young (2–3 months of age), WT aged (15–18 months of age) and iMycTg aged-E (15–18 months of age) mice were infected with 10 cysts of Toxoplasma gondii and serum was collected after euthanasia on day 9 post-infection for cytokine measurement (Eve Technologies, Calgary, AB, see Materials and methods). Serum samples from uninfected WT young mice were used as an additional control. iMycTg mice were analyzed on average 4.2 months after induction (range: 3.7–5.1 months). *p < 0.05, **p < 0.01 and ***p < 0.001. The data underlying this figure can be found in S7 File. (TIFF) [file pbio.3003283.s008.tiff]

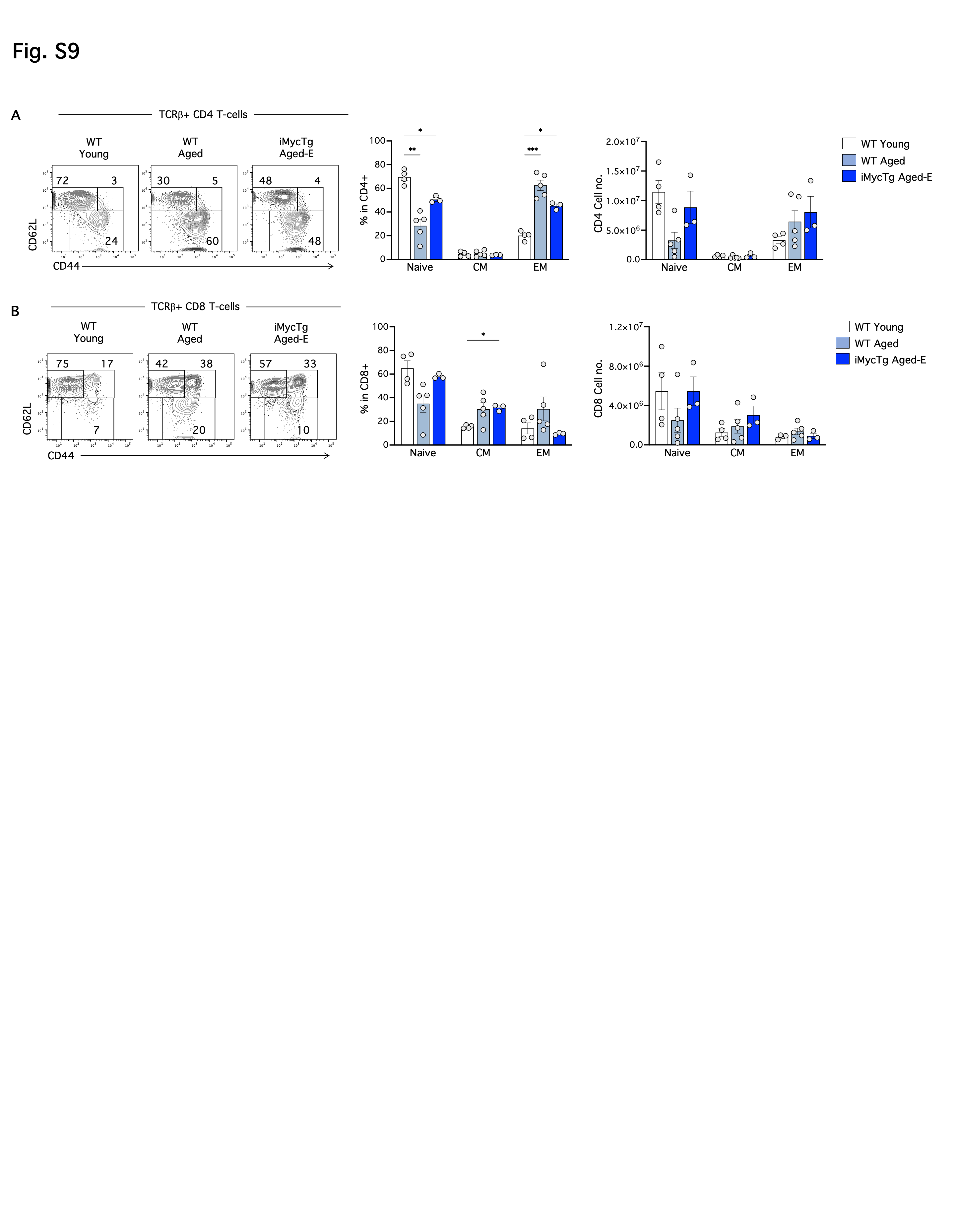

Supplement: S9 Fig — Representative FACS plots showing CD62L and CD44 expression in (A) TCRβ+ CD4 T-cells and (B) TCRβ+ CD8 T-cells in WT young (2–3 months of age), WT aged (15–18 months of age) and iMycTg aged-E (enlarged) (15–18 months of age) mice, 7 days post T. gondii infection. Bar plots depict percentages and numbers of CD62L+ CD44− (naive), CD62L+ CD44+ central memory (CM) and CD62L− CD44+ effector memory (EM) in the indicated mouse groups. Mice were infected 3.6 months after induction. *p < 0.05, **p < 0.01, and ***p < 0.001. The data underlying this figure can be found in S7 File. (TIFF) [file pbio.3003283.s009.tiff]

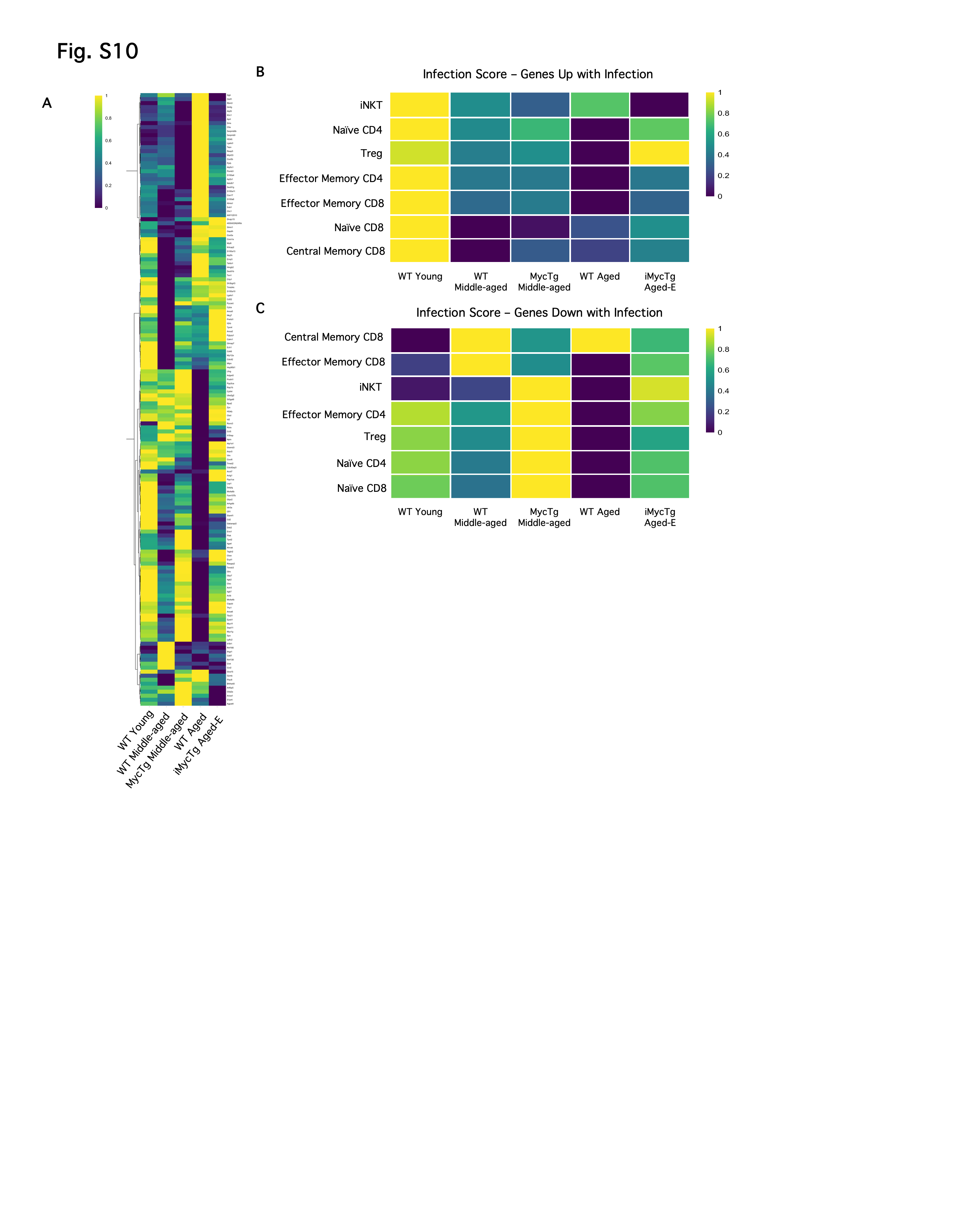

Supplement: S10 Fig — Mice were infected with T. gondii and sacrificed on day 12 post-infection for scRNA-Seq analysis on splenic T-cells (refer to Fig 9). (A) A scaled heatmap showing the expression of genes previously identified as being associated with the Th1 response [63]. (B) and (C) Scaled heatmaps displaying infection scores generated using genes (B) upregulated or (C) downregulated in infected WT young samples compared to WT young (2–3 months of age) steady state samples per indicated T-cell cluster, per individual mouse group listed. The data underlying this figure can be found in S7 File. (TIFF) [file pbio.3003283.s010.tiff]
